# Implications of Polishing Techniques in Quantitative X-Ray Microanalysis

**DOI:** 10.6028/jres.107.052

**Published:** 2002-12-01

**Authors:** Guy Rémond, Clive Nockolds, Matthew Phillips, Claude Roques-Carmes

**Affiliations:** Australian Key Centre for Microscopy and Microanalysis, The University of Sydney, NSW 2006, Australia; Laboratoire de Microanalyse des Surfaces, Ecole Nationale de Mécanique et des Microtechniques, Besançon, France; Electron Microscope Unit, The University of Sydney, NSW 2006, Australia; Microstructural Analysis Unit, University Technology of Sydney, NSW 2007, Australia; Laboratoire de Microanalyse des Surfaces, Ecole Nationale de Mécanique et des Microtechniques, Besançon, France

**Keywords:** abrasive wear, bound abrasives, chemical-mechanical polishing, loose abrasives, polishing, surface and subsurface damage, x-ray microanalysis

## Abstract

Specimen preparation using abrasives results in surface and subsurface mechanical (stresses, strains), geometrical (roughness), chemical (contaminants, reaction products) and physical modifications (structure, texture, lattice defects). The mechanisms involved in polishing with abrasives are presented to illustrate the effects of surface topography, surface and subsurface composition and induced lattice defects on the accuracy of quantitative x-ray microanalysis of mineral materials with the electron probe microanalyzer (EPMA).

## 1. Introduction

An optically flat polished surface is a necessary criterion satisfying the geometrical conditions for quantitative x-ray microanalysis with the electron probe microanalyzer (EPMA). All mechanical, structural, physical and chemical surface modifications resulting from the surface preparation will affect the accuracy of quantitative x-ray microanalysis as previously reported by Rémond [1]. The objective of this presentation is to convey to the EPMA community that polishing with abrasive particles is a complex operation involving many experimental and instrumental factors that are characteristic of the materials to be polished. For this purpose, the mechanisms involved in abrasive wear will be presented in order to illustrate some consequences of the polishing procedure on the reliability of quantitative x-ray microanalyses.

Mechanical polishing is performed by means of abrasives with decreasing grain size until scratches are no longer visible (optically polished surface). From a mechanical point of view, during the first stage of preparation, coarse grains are used to remove initial surface topographical and chemical defects. The next stage with smaller abrasive grain size aims to obtain the final quality of the surface satisfying the conditions for EPMA analysis. This regime is often divided into two operations, e.g., the intermediate polishing and the final polishing.

**Fig. 1:**
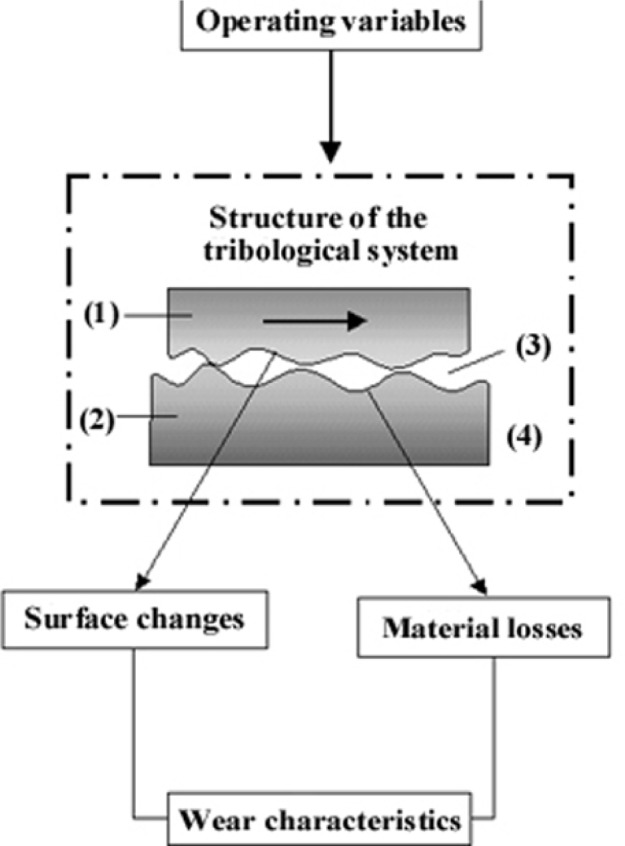
Schematic representation of a tribological system according to the norm DIN 50 320. The tribological system consists in (1) the specimen to be polished, (2) the abrasive specimen, (3) the interfacial medium and (4) the surrounding medium.

The mechanisms involved in mechanical polishing using abrasive particles are part of tribology, the discipline studying material science, physics, chemistry and surface contact engineering [2–5]. A description of a tribological system (according the norm DIN 50 320) consists of a set of experimental parameters (applied load, velocity and duration of the motion) and the system structure (the two bodies in contact, the interfacial and surrounding media), as shown in Fig. 1.

**Fig. 2:**
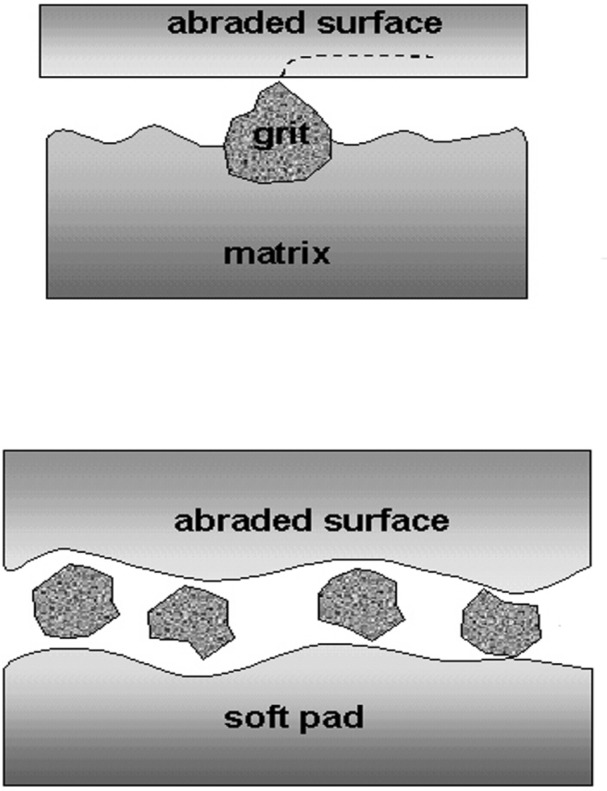
Abrasive wear in two body and three body configurations. a) two body situation with abrasives bound to the polishing substrate, b) three body situation resulting from wear debris or loose abrasives trapped in the interface between the specimen and a polishing pad.

Wear is defined as a cumulative surface damage phenomenon in which material is removed from a body as small debris particles, primarily by mechanical processes. The wear mechanism is the transfer of energy with removal or displacement of material. The four major wear mechanisms are adhesion, abrasion, surface fatigue and tribochemical reactions. In polishing with abrasive particles, the wear mechanism is mostly abrasive wear but other mechanisms are also possible. The abrasive mechanisms occurring in a dry or humid environment, result from the simultaneous actions of normal and tangential forces and are materialized by the development of ploughing grooves or scratches which are in some instances accompanied by hertzian fractures. For the classification of the abrasive wear modes, we will use the most widely accepted terminology known as two-body abrasion and three-body abrasion. This terminology illustrates the experimental situations encountered in the polishing techniques as illustrated in Fig. 2. In a two-body mode, the bound abrasive particle (identified as a guided-cutting tool) is solidly fixed to the substrate (Fig. 2a). In a three body abrasive mode, free (or loose) particles form a slurry between the specimen surface to be polished and a flat polishing substrate as illustrated in Fig. 2b. The free particles in a three-body wear mode may be intentionally added abrasives or be detached debris from the worn surface.

The manifestations of abrasive wear are the change of the surface roughness resulting from material removal and the change of the physical and chemical properties of the surface and subsurface with respect to those of the bulk. In addition to the mechanical and geometrical description, these deformations are accompanied by (i) the production of highly localized heat, (ii) the creation of excitations and defects in the material, (iii) the production of dangling bonds and trapped electrons, (iv) and the emission of excited and reactive species into the gas phase (exo-emission). All these phenomena result in a highly reactive surface accompanied by the formation of a surface composition different from that of the bulk. Separation of charges also leads to the creation of intense electric fields at the surface of many insulating materials.

When a single type of material is routinely analyzed with the EPMA, it is possible to optimize the polishing strategy, minimizing the thickness of the damaged surface. However, there is no general selection rule for the operating conditions because, for a given material, the wear mechanisms depend not only on the specimen properties but also on all of the tribological interactions between the abrasive materials and the specimen to be worn (see Fig. 1). Generally, most of the EPMA laboratories have to analyze a variety of specimens which often contain several different phases. Standards blocks also contain several materials and the polishing procedure cannot be optimized for all phases present in the heterogeneous material.

The consequences of the abrasive wear on the accuracy of quantitative x-ray microanalysis data are illustrated by the following examples: (1) chalcopyrite (CuFeS_2_) in a massive form and as inclusions in a silver sulfide matrix, (2) a binary quartz (SiO_2_)-arsenopyrite (AsFeS) mineral, (3) *α*-alumina crystals and (4) synthetic polycrystalline ZnS obtained by chemical vapor deposition.

## 2. Abrasive Wear

### 2.1 Two Body Abrasive Wear

**Fig. 3:**
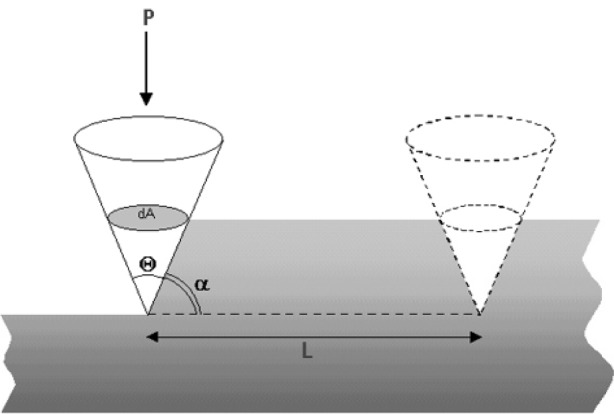
Model of abrasive wear by a conical shaped particle.

Models of abrasive wear with bound abrasives assume that the abrasive asperity is like a sharp tool producing a groove into a surface (Fig. 3).

The volume d*V* of material removed by an individual rigid cone shaped punch with a half-apex angle *θ* sliding at the surface of the specimen along a distance dL under an applied load d*P* is:

$$\frac{dV}{dL}=\frac{2\;\cot\;g\theta }{\pi }\cdot \frac{dP}{H}=\frac{2\;\tan\;\alpha }{\pi }\cdot \frac{dP}{H}$$ (1)

where *H* is the Meyer hardness of the indented material and *α* is the attack angle of the particle for which the sliding direction is parallel to the specimen surface.

The total volume *V* of removed material is the sum of volumes removed by each individual particle sliding along a total length L at the surface of the abraded specimen is expressed as:

$$\frac{V}{L}=K\cdot \frac{P}{H}$$ (2)

The above relation is identical to the experimental Archard’s law which is a general law describing the wear mechanisms [6].

The coefficient, *K*, is the wear coefficient i.e., the probability for an elementary volume of matter to be removed by an abrasive grain. The order of magnitude of the *K* value is a predictive signature of the wear mechanism. For the case of abrasive wear, *K* is about 10^−1^ and is lower than 10^−2^ for the case of adhesive wear.

The hardness, *H*, is not the unique parameter characteristic of the material involved in the abrasive wear and the toughness, the stiffness and the strain-hardening yield must also be considered. However, in practice, the hardness of the particles remains a criterion for the selection of the abrasives for polishing and it is commonly accepted that a particle with hardness *H*_a_ will scratch a surface with hardness *H*_s_ when *H*_a_ > 1.2 *H*_s_.

In the simplified model of abrasive wear, it is assumed that all the material is removed from the groove resulting from the sliding path. In fact, the groove may result from two extreme conditions, which are micro-cutting and micro-ploughing, respectively. An event in which 100 % of the displaced volume is removed in the form of chips (wear debris) is a micro-cutting event. An event in which zero primary removal occurs in a single pass of the particle is micro-ploughing. Multiple ploughing events over a region can also produce wear debris.

**Fig. 4:**
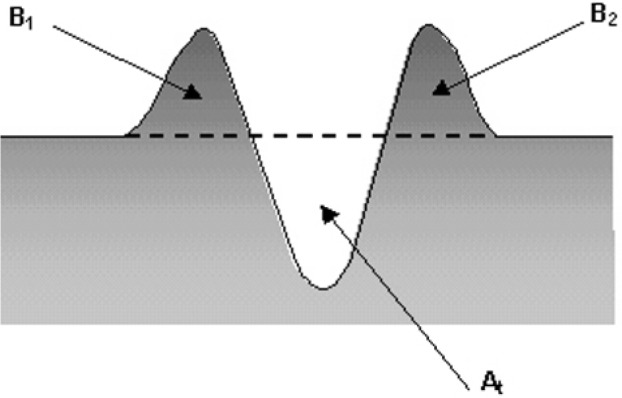
Vertical cross section of a groove induced by a particle sliding on the specimen surface: A_t_ is the total volume indented by the particle, B_1_and B_2_ correspond to the materials laterally displaced.

After a sliding abrasive action along a distance *L*, the rate of material removal *S*_ab_ is defined by:

$$S_{\text{ab}}=\left[A_{t}-\left(B_{1}+B_{2}\right)\right]/A_{t}$$ (3)

where *A*_t_ is the total area of the vertical cross section of the groove induced by the sliding particle, *B*_1_ and *B*_2_ are the cross section areas of the material displaced on each side of the groove as shown in Fig. 4. The wave dimensions depend on the shape and speed of the punch.

For a single scratch, the *S*_ab_ value is determined by the deformation mechanism. For example, with *S*_ab_ = 0 there is a plastic deformation without formation of debris whereas with the second extreme case with *S*_ab_ = 1 there is a micro-cutting process in which all the material is removed.

The wear mechanism is never purely micro-cutting or micro-ploughing and the proportion of both mechanisms will depend on the dimensions and the morphology of the abrasives, the hardness of the abrasives relative to that of the worn material and the mechanical behavior of the polished materials:

1. the abrasive wear rapidly increases with the size of the particles to reach a plateau value and,
2. the abrasive wear is a function of the relative hardness *x* = *H*_a_/*H*, where *H*_a_ is the hardness of the abrasives and *H* is the hardness of the material to be polished. The abrasive yield is low for *x* < 0.7, then linearly increases for 0.7 < *x* < 1.7, before reaching saturation when x > 1.7 and,
3. there is a critical angle for the particles leading either to the formation of a plastic deformation with formation of a groove or formation of a scratch with removal of material [7]. Micro-cutting will be the dominant mechanism for an attack angle (*α*) greater than the critical angle (*α*c), while micro-ploughing will occur at *α* < *α*c.

The transition from micro-ploughing to micro-cutting is also observed for the case of indenters, which are characterized by *α* ≪ π/2 as for a spherical punch describing a worn abrasive particle. Elastic and elastic-plastic strain modes are possible. When the applied load is increased, elastic and plastic deformations successively occur before the material is pushed ahead of the particle and removed in a series of flat chips. This mechanism is known as wedge and fin formation.

The transition from the micro-ploughing to the micro-cutting does not only depend on the geometrical characteristics of the abrasives but the rheological behavior of the materials must also be considered, i.e., the evolution of strains and the flow of materials exposed to an applied load.

The contact mechanics provides a description of the stresses and strains at the surface in comparison to those in the bulk material. When the abrasive particle is worn, it is usual to describe it as a spherical shaped punch.

The Hertz theory shows that for the case of a contact between a spherical punch of radius *R* and a plane surface, the radius, *a*, of the circular contact area under a normal applied load *P* (static indentation) is *a* = *PR*/*K*, where the parameter *K* is a function of the Young modulus and the Poisson coefficient. The resulting penetration depth of the punch is

$$\delta =a^{2}/R$$ (4)

The analysis of the spatial distribution of stresses shows that the shearing stress maximizes at a depth below the specimen surface (Hertz point). When the applied load increases, i.e., when the penetration depth of the punch into the substrate increases, the material begins to be locally plastically deformed beneath the punch. The yielding stress threshold corresponds to *P*_m_ = 1.1 *σ*_0_ where *σ*_0_ is the yielding stress. The volume beneath the punch is fully plastically deformed when P = 2.8 *σ*_0_.

In practice, it is necessary to account for the geometry of the contact surface in order to determine the limit between an elastic and a plastic behavior of the materials as a function of the contact pressure. For this purpose, a plasticity index *Ψ* has been introduced to define the beginning of a plastic deformation [8]. This index is expressed by:

$$\Psi ={\left(\frac{E}{\left(1-v^{2}\right)H}\frac{\sigma }{\beta }\right)}^{1/2}$$ (5)

where *E* is the Young modulus, *σ* is the standard deviation of the distribution of the height of the surface asperities and *β* is the mean radius of the asperities. It is accepted that for *Ψ* < 0.6, the contact is elastic and for *Ψ* > 1, the contact is plastic.

**Fig. 5:**
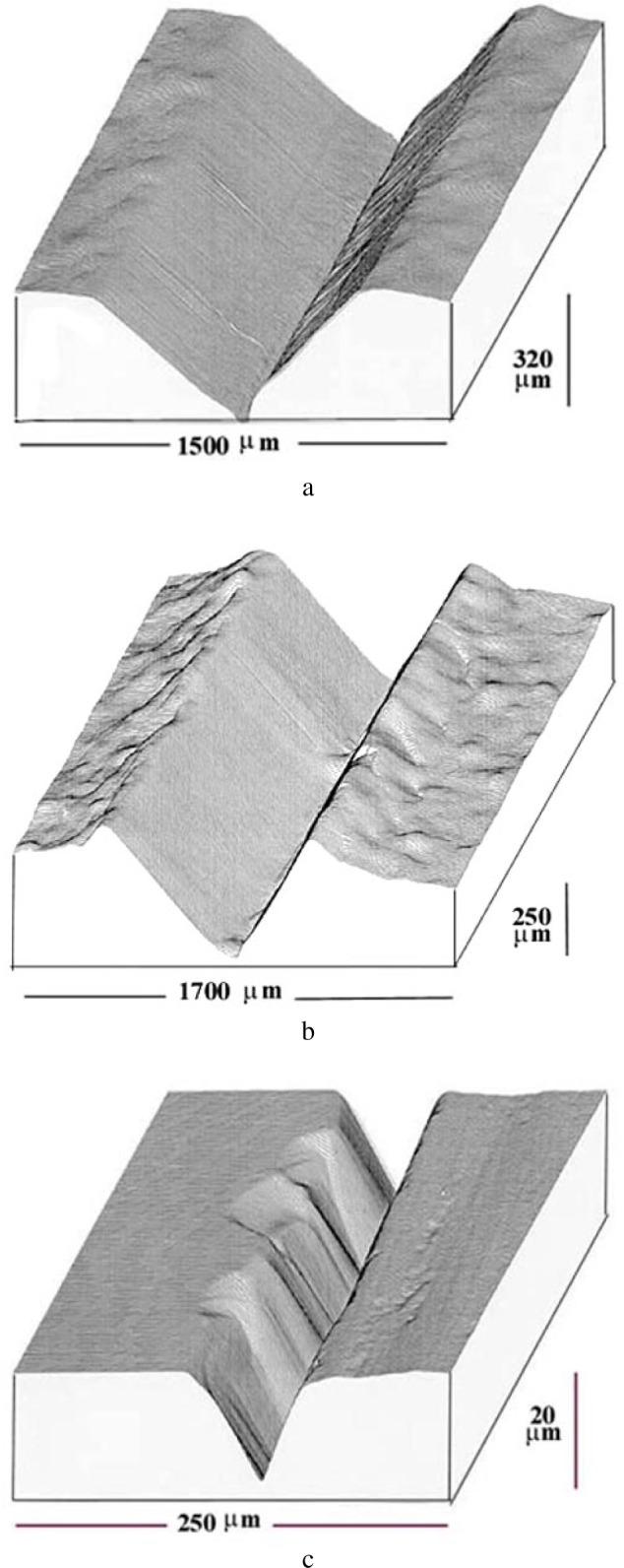
Shapes of the abraded volumes by a sharp particle as a function of the abrasive wear mechanisms shown by scanning mechanical microscopy a) micro-cutting process with material removal, b) plastic deformation with lateral displacement of material without debris removal and c) micro-fracture process.

**Fig. 6:**
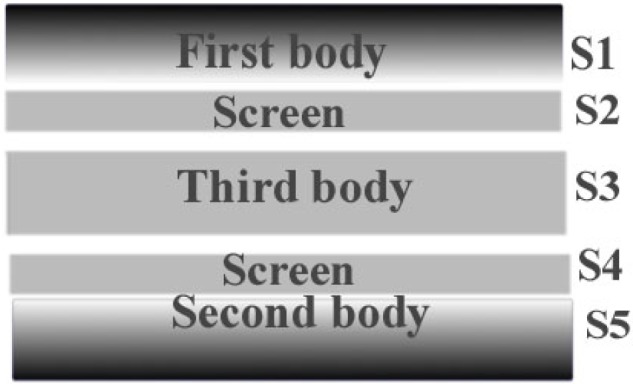
Velocity accommodation model according to Berthier [11].

For a dynamical regime, i.e., when the bound abrasive particles is sliding on top of the surface, the sclerometry provides a description of the mechanisms associated with the abrasive displacements relative to those of the material to be polished. The three dimensional cartographies in Fig. 6 using a scanning mechanical microscope described in Ref. [9], illustrate the shape of the groove resulting from micro-cutting (Fig. 5a), and from plastic deformation (Fig. 5b). In brittle materials, tensile stress can lead to cracks that propagate and fragments of material can be removed (Fig. 5c).

According to the model proposed by Bowden and Tabor [9], the tangential load Ft necessary to move a cone shaped tip is the sum of two terms. The first term characterizes the friction and the second one characterizes micro-ploughing. The model has been extended by Gauthier and Schirrer [10] to the strain mechanisms, the depth of an elastic-plastic scratch can being expressed as the sum of a depth resulting from an elastic sliding and a depth resulting from a ploughing action.

### 2.2 Three Body Abrasive Wear

A three-body abrasive wear situation occurs when loose particles can move in the interface between the specimen surface and the polishing wheel. Such a situation occurs when abrasives are intentionally deposited and can roll on top of the polishing substrate as will be illustrated for the case of the final polishing step with very small grains on soft lubricated pads. A three body mode may also occur when small pieces of material are detached from the specimen to be polished and become trapped or circulate within the contact between the two first bodies. These wear debris associated with superficial tribological transformations (STT) result in the formation of a third body which can be ejected from the contact or can circulate in the interface between the specimen surface and the polishing wheel. The composition of the third body is usually different of that to the first body from which they are detached and can be described as a core with outer screens [11]. The composition of the screens results from tribological reactions of the core with the outer surfaces of the first two bodies. From the kinematics point of view, the third body is an operator that transmits the applied load from the first to the second body and accommodates the difference of velocity between the two bodies. In order to account for these interactions, the notion of velocity accommodation sites has been introduced by Berthier [11]. These accommodation sites are labeled S_1_ to S_5_ as illustrated in Fig. 6. Each site can accommodate the velocities according to four mechanical modes: an elastic mode (M_1_), a tribological failure (M_2_), a shearing mode (M_3_) and a rolling displacement of the third bodies within the contact (M_4_). Combining the five accommodation sites and the four accommodation mechanical modes leads to 20 accommodation mechanisms in the contact resulting in a more complex case than the two body abrasive wear.

The velocity accommodation mechanisms between the surface to be polished and the third bodies are of the type S_1_ M_1,2,3,4_ or S_2_ M_1,2,3,4_ for the case of the screens of wear debris interacting with the surface from where they are detached and of the type S_3_ M_1,2,3,4_ for the case of loose abrasives.

The velocity accommodation mechanisms between the polishing wheel and the third bodies are of the type S_5_ M_1,2,3,4,_ S_4_ M_1,2,3,4_, or S_3_ M_1,2,3,4_.

In a recent review on polishing in free abrasive conditions on a soft pad, Xie and Bhusham [12] established a linear relationship between the wear rate and a dimensionless abrasive index defined by:

$$\frac{P}{E_{P}}{\left(\frac{R}{\sigma }\right)}^{0.3}\;{\left(\frac{H_{P}}{H_{w}}\right)}^{1.5}$$ (6)

where *P/E*_p_ determines the contact area between the specimen surface and the polishing substrate where, *P* is the contact pressure and *E*_p_ is the elastic modulus of the polishing pad. *R* is the radius of the abrasive particle (assumed to be spherical), *H*_p_ and *H*_w_ are the hardness of the surface to be worn and of the polishing pad, respectively. In the above expression of the wear index it is assumed that *H*_p_ is smaller than *H*_w_ and that the hardness of the abrasive is higher than *H*_p_ and *H*_w_. The parameter *σ* is the standard deviation of the height distribution of the polishing pad.

A three body wear situation is more generally representative of the final polishing stage consisting of free small abrasive particles dispersed in a suspension between the specimen surface and a polishing pad. These free abrasives are able to roll on the surface of the polishing pad so that the abrasives (or the particles detached from the specimen surface) tend to be orientated along their longest dimension leading to small attack angle with respect to the specimen surface. As a result, the shape of the particles has less effect on the wear rate for the case of free abrasives than for bound abrasives. Under the applied load some particles can segregate and be embedded into the pad resulting in a cutting action of the abrasives with the production of scratches. The hardness and roughness of the polishing pad also influence the wear rate in a free abrasive situation, since increasing the load applied to the specimen surface does not strongly increase the contact pressure on each individual abrasive particle.

## 3. Polishing Procedures and Techniques

The sample of interest is first embedded in a resin or a metallic alloy. When resins are used, metallic powder can be added to the resin before polymerization in order to increase the electrical conductivity. In practice, the specimen preparation includes the following steps: polishing with coarse abrasives (grinding), intermediate and final polishing. Some of the frequently used polishing procedures are summarized in Table 1.

### 3.1 First Stage: Polishing With Coarse Abrasives

The specimen is first polished with coarse abrasives in order to have a flat surface and to reduce the amount of deformation induced by the sectioning operation. The polishing material can be an emery paper (bound abrasive process) or carbide abrasives in a slurry deposited on top of a glass or a cast-iron plate (loose abrasive process).

### 3.2 Second Stage: Intermediate Polishing

**Fig. 7:**
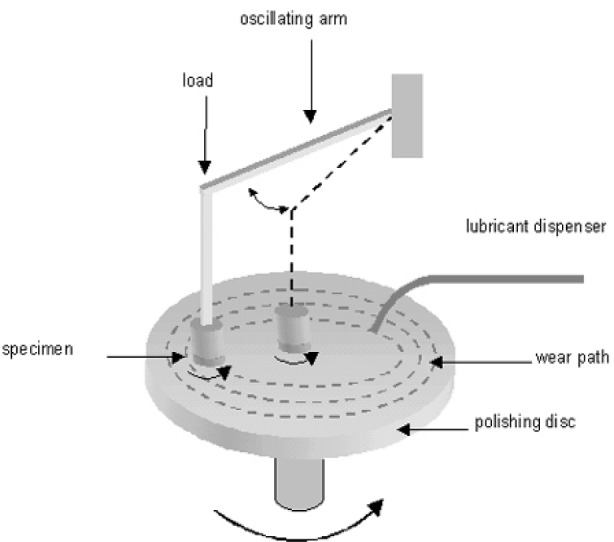
Schematic description of a polishing equipment.

The intermediate polishing can be manually performed using emery paper of decreasing grain size. More frequently this operation is performed with automated polishing equipment allowing the simultaneous preparation of several specimens. A polishing instrument (Fig. 7) consists of a metallic rotating abrasive wheel holding the specimen surface to be worn. Some polishing discs have wearing paths separated by wide grooves in which the wear debris can be trapped. Under the combined actions of the rotation and the applied load, the specimen can spin on top of the polishing disc. The experimental factors controlling the wear rate and the wear mechanisms are the applied load, the rotation speed of the polishing wheel, the polishing duration, the size and the shape of the abrasive grains and the nature of the lubricant. Typically, the applied load to the specimen ranges from 0.5 kg to 1 kg. With some polishers, an arm holding the specimen can sweep horizontally so that the specimen is moved along the radius of the polishing substrate. A dispenser provides a lubricant during the polishing procedure. With automated equipment all experimental conditions can be adjusted at any time of the polishing sequences and are reproducible for repetitive polishing operations.

The intermediate polishing sequence is frequently performed with diamond or alumina particles dispersed in a paste or in a liquid on a soft metallic plate (such as tin) or on a hard cast iron plate covered with a foil of a soft metal such as aluminum. The abrasives penetrate into the soft metallic disc resulting in a bound abrasive operation. Decreasing the grain size of the abrasive particles aims to remove the damage resulting from the previous step. However it must be kept in mind that each successive polishing step creates its own damage. It is recommended to progressively decrease the dimensions of the abrasives, the most frequently successive sequence consisting of 6 μm, 3 μm, and 1 μm abrasive particles. Our recent observations in polishing alumina crystals show that if the polishing step with 3 μm particles is omitted, residual damage created by the 6 μm abrasives still exists after the 1 μm polishing procedure is applied.

### 3.3 Third Stage: Final Polishing

The final polishing operation aims to eliminate residual scratches and to remove surface compounds formed during the previous intermediate polishing steps and to make visible precipitates that can be hidden by the remaining damaged surface layer.

The first step in the final polishing is a mechanical polishing with fine abrasives (typically 0.5 μm or 0.25 μm) on a hard polishing cloth. Hard polishing cloths have faster polishing rates than soft cloths and less relief at interfaces with different hardness but tend to produce a severely damaged surface. For this intermediate polishing sequence diamond in a suspension of oil or a paste is commonly used. Alpha alumina (hexagonal structure) in the form of powder with water as a carrier is also used. In some laboratories, an additional polishing is performed on a soft pad with abrasives less than 0.05 μm with water as carrier. Other common abrasives are γ-alumina (cubic structure), magnesium oxide, chromic oxide or cerium oxide powders.

For metals, electrolytic or specific chemical etching can be used to reveal structures. For insulators, chemically assisted mechanical polishing (CMP) is used combining chemical reactions with soft wear abrasion. For this purpose, the polishing suspension composed of a basic liquid (pH = 10) and colloidal SiO_2_ as abrasive (typically 20 nm in size) is used on a soft polishing pad (three body polishing system). Polishing with colloidal silica results from a combination of mechanical and chemical action as discussed by Liang et al. [13]. For metals, the CMP polishing is a two step mechanism consisting in the formation of an oxide layer which is progressively removed by mechanical abrasion. In the CMP polishing process, the loose abrasive wear induces cutting and ploughing at a nanometer-scale. However, the abrasive wear may produce rounded edges on the polished material. Reaching the geometrical quality requires a careful attention to the quality of the soft polishing pad which also suffers abrasive wear during the polishing procedure as discussed by Byrne et al. [14]. It is recommended to frequently renew the pad and to keep it always wet in order to avoid the risk of crystallization of the colloidal silica that can produce scratches.

## 4. Characterization of Polished Surfaces

The two polishing procedures described in Table 2 were used to prepare the polished surfaces.

### 4.1 Surface Topography

The topography of polished surfaces as a function of the grain size of the abrasives was observed by scanning electron microscopy (SEM) and stylus based techniques such as scanning mechanical microscope (SMM) and atomic force microscope (AFM).

**Fig. 8:**
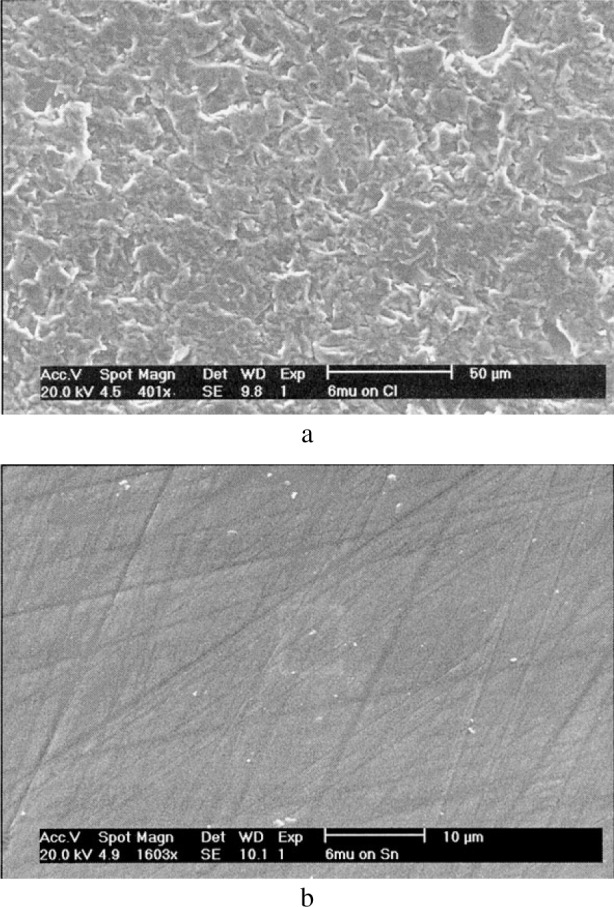
Effect of the nature of the polishing substrate on the surface topography polished with the same 6 m diamond grains and the same load applied on (a) a cast iron polishing wheel (loose abrasive process) and (b) a tin polishing wheel (bound abrasive process).

For given types of abrasives, the choice of the polishing substrate will determine the wear conditions, i.e., bound or loose abrasive wear and consequently will modify the surface topography as illustrated in Fig. 8. An *α*-alumina crystal was polished with a diamond paste (6 μm abrasive grain size) on a cast iron polishing wheel and on tin polishing wheel. After polishing on cast iron, the secondary electron image (Fig. 8a) shows that the surface topography consists of a high density of craters indicating that the wear mechanism is mostly ploughing. As shown in Fig. 8b for the case of polishing on a tin plate, scratches are visible indicating a cutting action of the abrasives that are bound to the polishing substrate.

The variations of the surface roughness as a function of the dimensions of the abrasive particles are illustrated for the case of a specimen consisting of two minerals with different hardness. The specimen consists of a quartz (SiO_2_)-arsenopyrite (AsFeS) assembly. The specimen was polished according to the procedure B in Table 2. The final polishing was alumina on a soft pad.

**Fig. 9:**
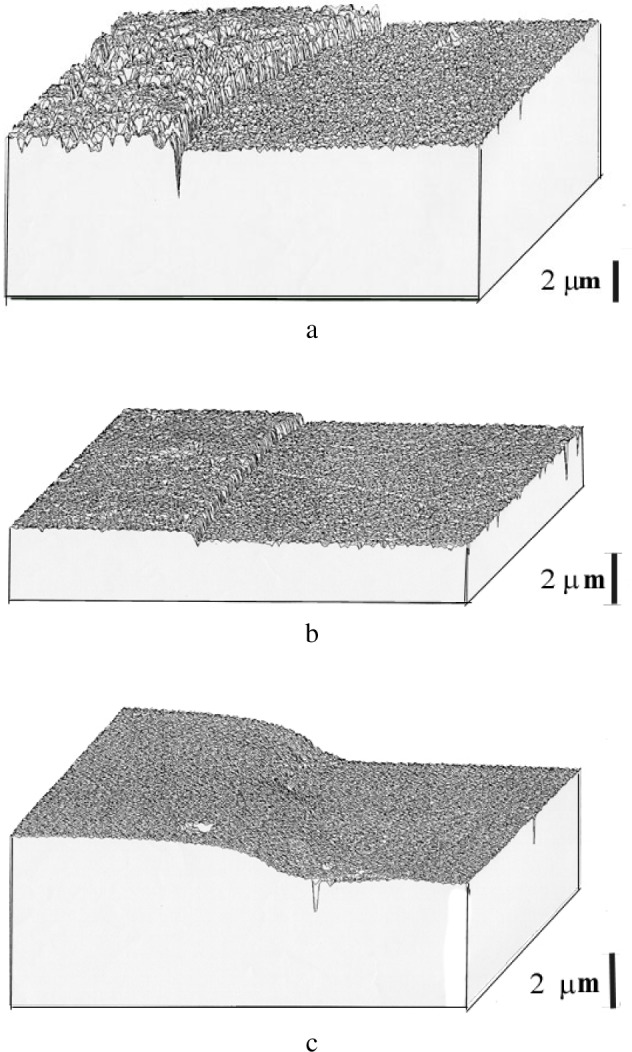
Scanning mechanical microscopy of quartz/arsenopyrite binary compound polished with (a) 6 μm bound diamond abrasives, (b) 1 μm bound diamond abrasives and (c) with loose alumina particles on a soft pad.

The 3-dimensional topography maps in Fig. 9b show that after a 6 and 3 diamond abrasive polishing successively, the surface roughness is higher for the quartz crystal than for the arsenopyrite mineral. In addition, a step is observed between the two surfaces indicating that the abrasive wear for arsenopyrite is higher than that for quartz consistent with the difference in hardness of the two adjacent materials [Archard’s law in Eq. (2)]. The variations of the surface topography for decreasing abrasive size can be characterized by the roughness criteria derived from 2D or 3D topography line scans or maps.

After a final polishing with alumina particles on a soft pad (loose abrasive process), the roughness of both surfaces (Fig. 9c) becomes smaller than the depth resolution of the SMM observation imposed by the 1 μm radius of the stylus used (the lateral resolution is equal to the stylus radius and the vertical resolution is Δ*z/z* = 10^−3^ where *z* is the altitude). The difference in height between the two polished surfaces is still clearly visible but the sharp edge becomes rounded after the abrasive polishing procedure with loose abrasive on a soft pad.

The effect of the final polishing procedure, i.e, purely mechanical versus CMP, is illustrated for the case of a synthetic polycrystalline ZnS specimen. Two final polishing procedures were applied: (1) 0.5 μm diamond abrasives on a napless nylon pad followed by polishing with alumina particles on a soft pad and, (2) polishing on a soft pad with colloidal silica spheres (20 nm in diameter) dispersed in solution with ph = 10, resulting in a chemical-mechanical polishing process.

The polished ZnS surfaces were examined by scanning electron microscopy in a variable pressure SEM (VP-SEM) in order to avoid surface coating and by AFM (contact mode).

**Fig. 10:**
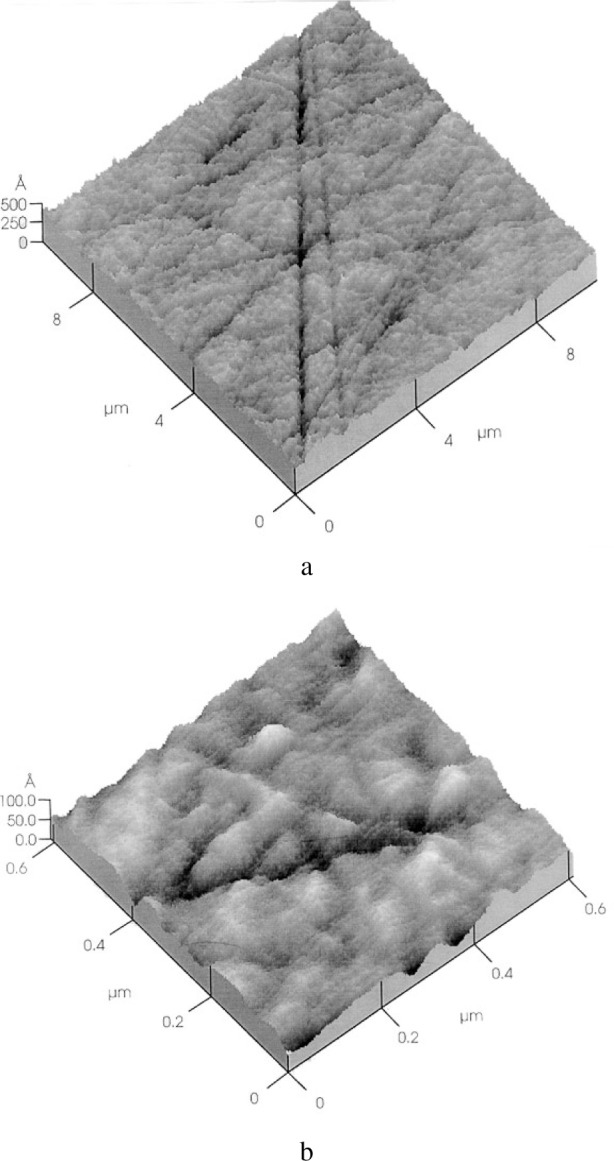
Residual surface topography shown by AFM imaging (contact mode) of a polished ZnS specimen. (a) 10 μm × 10 μm sampled area and (b) 0.6 μm × 0.6 μm area similar to the electron beam spot size with the EPMA.

**Fig. 11:**
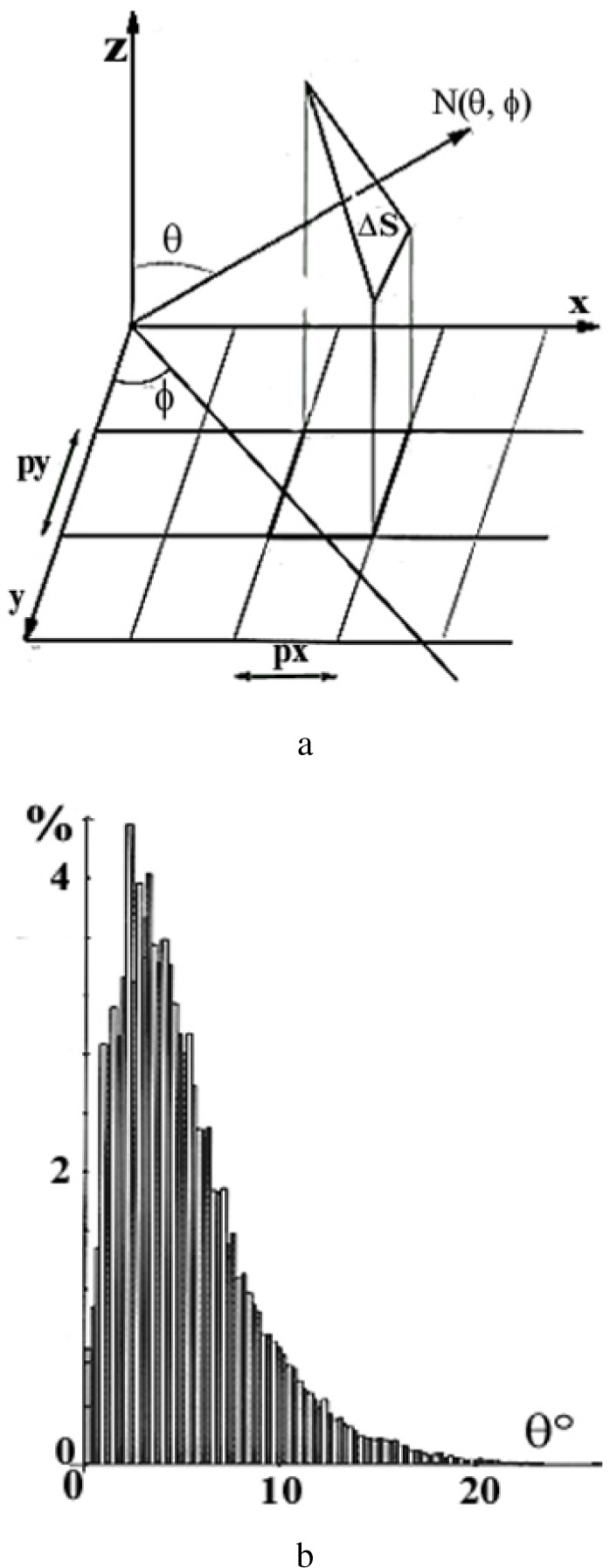
Geometrical processing of surface topography. a) digital decomposition of the rough surface in elementary triangles and b) distribution *P*(*θ*,*φ*) of the number of elementary triangles measured from the geometrical processing of the AFM image in Fig. 12.

As shown in Fig. 10, AFM images taken from the alumina polished ZnS specimen, a high density of scratches of small depth and width are still present. The total surface roughness derived from the AFM images is in the order of magnitude of 0.03 μm. In order to have an analytical description of the surface topography, the AFM images were decomposed into the sum of elementary triangles as described by Reemond et al. [15]. Each triangle is characterized by the direction of its normal (Fig. 11a). The distribution *P* (*θ*, *φ*) expresses the number of normal directions i.e. the number of elementary triangles corresponding to an orientation (*θ*, *φ*) of the micro-facets describing the surface topography (Fig. 11b).

**Fig. 12:**
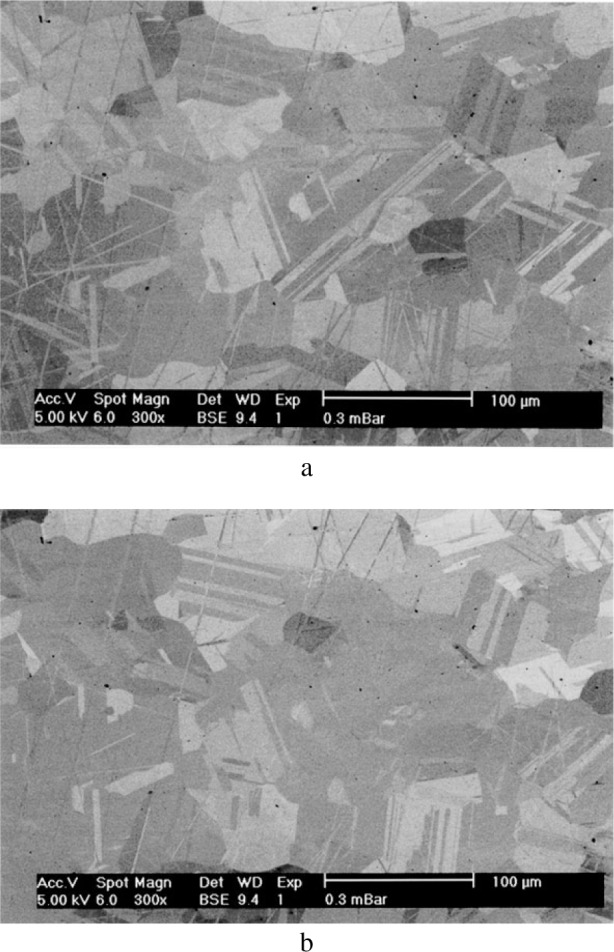
Backscattered electron image of an uncoated mechanically-chemically polished ZnS specimen on a soft pad with colloidal silica particles dispersed in a solution of pH = 10. (a) 0° tilt angle and (b) 2° tilt angle.

**Fig. 13:**
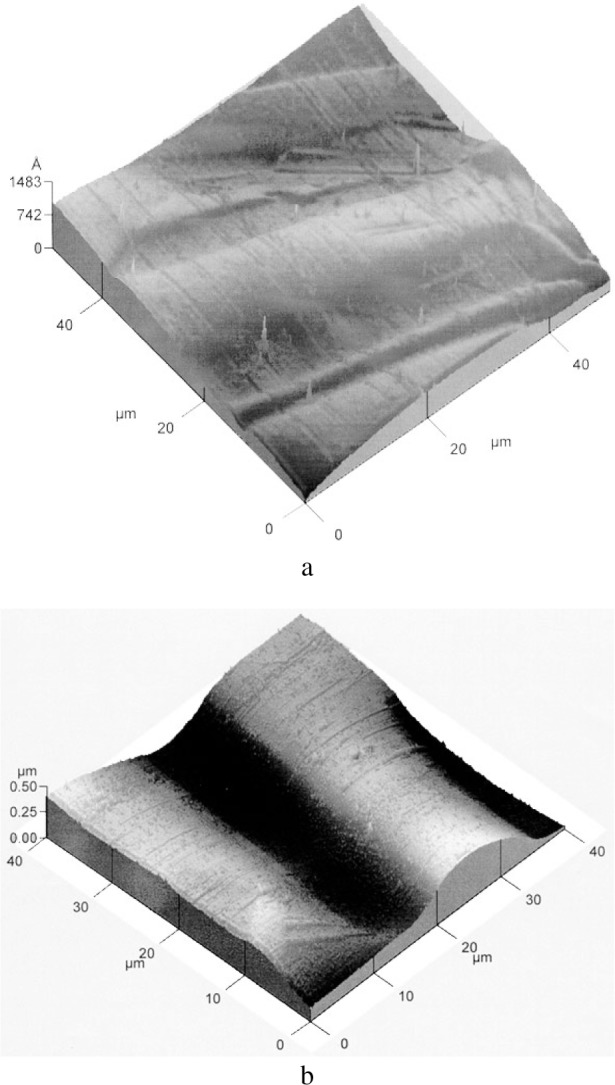
Surface topography shown by AFM imaging (contact mode) of a polished polycrystalline ZnS specimen polished with colloidal silica particles. Note the rounded edges of the crystallites.

An example of a CMP polished surface using colloidal silica spheres is shown on the backscattered electron image in Fig. 12 for the case of the synthetic ZnS specimen. Areas of a few tens of microns in size with different brightness are revealed while no such features were observed at the surface of the mechanically polished surface with alumina powders. The contrast of the BSE image varied as a function of the accelerating voltage and as a function of the tilt angle suggesting that the observed features correspond to different crystallographic orientations of the ZnS crystallites. As shown in the AFM images of the chemical-mechanical polished ZnS surface (Fig. 13), the crystallites exhibit differences in altitude due to a variation in hardness as a function of the crystal orientations. In addition the edges of the crystals were rounded.

### 4.2 Surface Versus Volume Composition

Examples of surface composition modifications with respect to that of the bulk composition as a function of the polishing procedures are illustrated for the case of a chalcopyrite (CuFeS_2_) mineral in a massive form and as inclusion in a natural acanthite (Ag_2_S) matrix.

#### 4.2.1 Massive Specimens

A massive chalcopyrite specimen was prepared according to the intermediate polishing procedure B in Table 2. After a final polishing with 0.5 μm diamond grains on a hard nylon pad, the surface was characterized by a brownish color. The surface was polished again on a soft polishing cloth using alumina powder dispersed in distilled water. The final polished surface exhibited a higher reflectivity than that of the diamond polished surface.

**Fig. 14:**
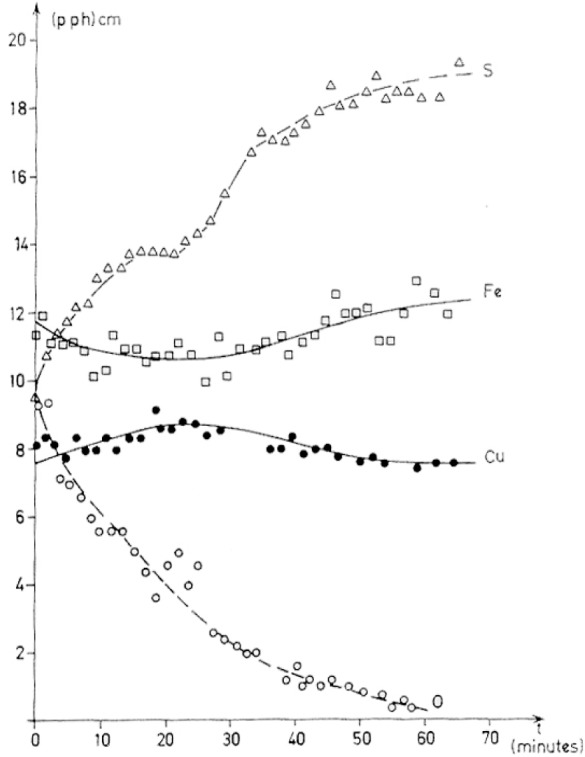
AES depth profile measured from a polished chalcopyrite (CuFeS_2_) specimen.polished with 0.25 μm diamond grains on a hard nylon pad.

Using surface sensitive techniques such as photoelectron spectroscopy (XPS-ESCA) and Auger electron spectroscopy (AES), and comparisons between experimental and calculated reflectance curves, Rémond et al. [16–19] showed that surface composition changes may occur immediately after polishing or after long exposure of the specimen to air. Below an outer carbon bearing contamination layer, the presence of sulfate and reduced sulfide compounds were detected at the surface of the diamond polished surface. Only iron was bound in the sulfate. AES sputter profiles in Fig. 14 show that the copper is rejected from the surface to form a copper-rich compound underlying the sulfate/oxide layer. The total thickness of the surface compounds was estimated to be <10 nm based on depth profiling. This thickness value agreed well with that derived from comparisons between experimental and calculated reflectance curves assuming the surface of chalcopyrite is covered with compounds identified by electron spectroscopies.

After the diamond polished chalcopyrite was polished again either with alumina (or chromic oxide) powders, the outer iron sulfate layer was no longer detected resulting probably from mechanical removal and/or from dissolution in the water used as a carrier of the abrasives. The iron oxide and copper rich layers were thinned after the final polishing on a soft pad leading to in an increase of the optical reflectivity of the chalcopyrite surface.

After exposure to the atmosphere during a few days or weeks, the polished chalcopyrite surfaces exhibit colored areas corresponding to an increase in thickness of the surface oxidized layer. Frequently these colored areas developed around holes or fissures and are associated with an increase in thickness of the iron oxide surface layer.

#### 4.2.2 Inclusions

Chalcopyrite inclusions with dimensions varying from a few tens to a few hundreds of micron in a silver sulfide (Ag_2_S) were analyzed. During the specimen preparation, some of the inclusions have been isolated from the silver sulfide matrix and remained embedded in the plastic mounting. Immediately after polishing all chalcopyrite inclusions exhibited a lower reflectivity than that of a freshly polished massive chalcopyrite. As reported by Reemond et al. [16], AES analyses showed that the freshly polished chalcopyrite surfaces were contaminated by C, O, and Ag bearing compounds.

**Fig. 15:**
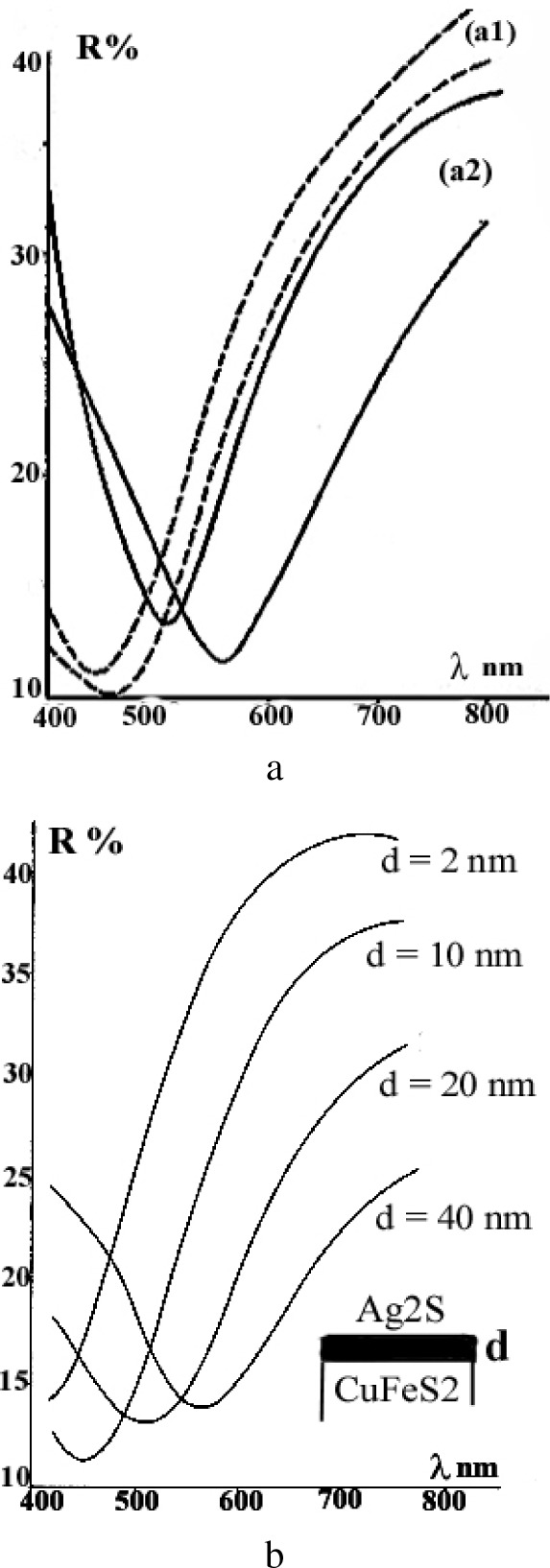
Optical reflectance curves from polished chalcopyrite inclusions in a silver sulfide matrix. (a) experimental curves measured (a1), a few hours after polishing and (a2), after illumination with an arc light source and, (b) calculated reflectance curves by varying the thickness of an Ag_2_S surface layer on top of the chalcopyrite substrate.

After the specimen was kept in the air during a few days, the reflectivity of the chalcopyrite inclusions isolated from the silver sulfide matrix decreased while the color of the inclusions in contact with the matrix turned to an orange hue. The tarnishing of the inclusions was accelerated by exposure of the specimen to an arc light. The optical properties of the chalcopyrite inclusions in contact with the silver sulfide matrix continuously varied as a function of time after the light illumination was turned-off. Changes in the optical properties of the chalcopyrite inclusions in contact with the silver sulfide matrix are illustrated by the experimental reflectance curves in Fig. 15a. These variations are consistent with an increase of the thickness of a silver bearing compound on top of the chalcopyrite inclusions as shown by the calculated reflectance curves in Fig. 15b.

### 4.3 Structural Disorders and Subsurface Lattice Defects

Synthetic polycrystalline ZnS specimens were used in this study. One specimen (labeled “clear ZnS”) consists of 20 μm to 35 μm crystallites and with a resistivity greater than 10^13^ Ω cm. The second specimen (labeled “yellow ZnS”) has crystallites 2 μm to 8 μm in dimensions and a resistivity of about 10^12^ Ω cm.

Diffraction patterns measured from the polycrystalline specimens after using a final CMP polishing procedure (see Fig. 12) indicated that the observed contrasts result from crystallographic effects rather than from variations in thickness of surface contaminants or chemical reaction products formed during the combined chemical-mechanical wear. Conversely, no similar crystallographic orientations were observed from SEM imaging of polycrystalline ZnS specimen using fine diamond or alumina particles for the final stage of polishing on a nylon or soft pad. No diffraction patterns could be measured from the mechanically polished ZnS surfaces suggesting the presence of an amorphous or a heavily damaged layer.

Surface physical properties changes of mechanically polished ZnS specimens were investigated by hardness measurements derived from Vickers micro-indentation performed under a 2 g and 500 g load, successively. The dimensions of the indented volumes were measured from AFM images of the crater induced by a 2 g load and by scanning mechanical microscopy (SMM) imaging for the case of a 500 g applied load. Calculated hardness values showed an increase of hardness near the surface [1].

**Fig. 16:**
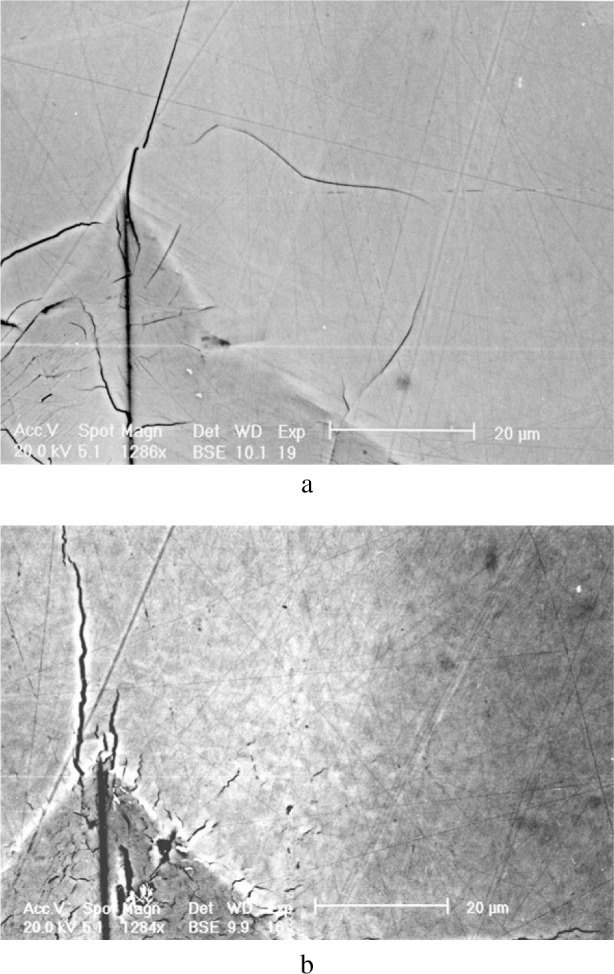
Secondary electron image of a polycrystalline ZnS specimen showing the propagation of fissures induced by a 500 g load Vickers indentation. (a) clear ZnS specimen and (b) yellow colored specimen.

Scanning electron imaging around the 500 g load indentation of the clear ZnS specimen with crystallites approximately 30 μm in size (Fig. 16a) showed fissures revealing the grain boundaries. For the case of the yellow ZnS specimen with a higher hardness but lower grain size than for the clear specimen, the crystallites are revealed on the secondary electron image (Fig. 16b) in the immediate vicinity of the indent. At a distance of about 30 μm to 40 μm from the edge of the indent the crystallites are no longer visible. In addition to the textural modifications, strains resulting in lattice disorders also develop under the static applied load as shown by cathodoluminescence studies [1].

Cathodoluminescence (CL) spectroscopy and microscopy in an SEM provides spatially resolved analysis of very small amounts of impurities and point defects responsible for energy levels located in the forbidden band gap in the energy diagram of the crystal. A review of the instrumentation and experimentation in the applications of CL to the characterization of mineral materials has been given by Rémond et al. [20].

**Fig. 17:**
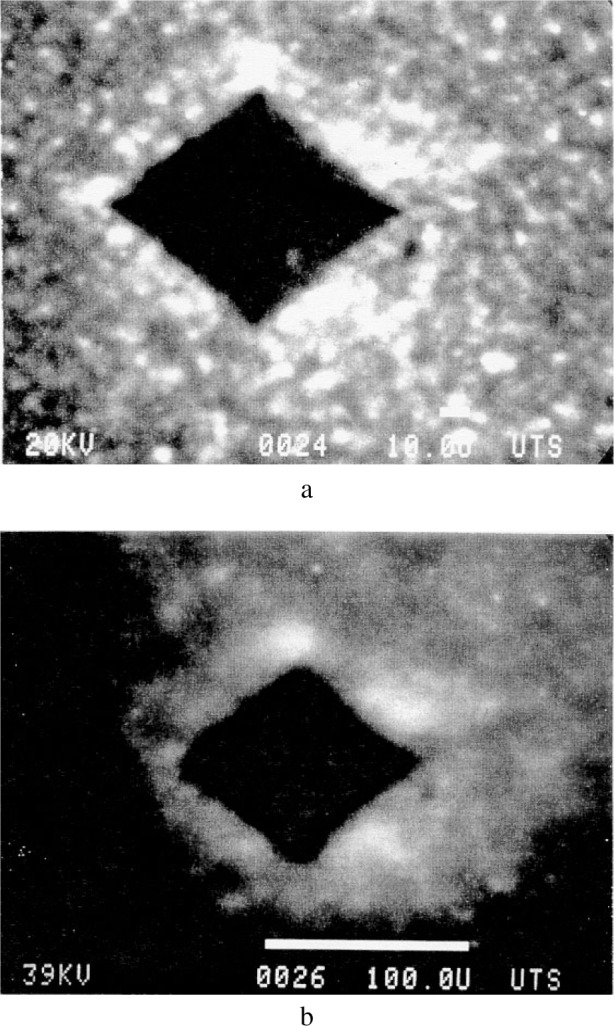
Panchromatic CL images around a 500 g load Vickers indentation of a polycrystalline ZnS surface (a) 20 keV and (b) 39 keV incident electron energy.

On the CL panchromatic image in Fig. 17a (spectrally unresolved CL emission), obtained with a 20 keV incident electron energy, the indented area appears dark. The absence of CL in the crater may result from plastic deformation annealing the CL emission or from an instrumental defocusing artifact due to the large depth of the indent below the specimen surface. Outside of the indent, the CL intensity exhibits bright spots. The highest density of bright CL spots occurs in a close proximity of the edges of the crater. Increasing the incident energy from 20 keV (Fig. 17a) to 39 keV (Fig. 17b), i.e., increasing the sampling depth, shows that deep subsurface strain fields developed during the indentation.

**Fig. 18:**
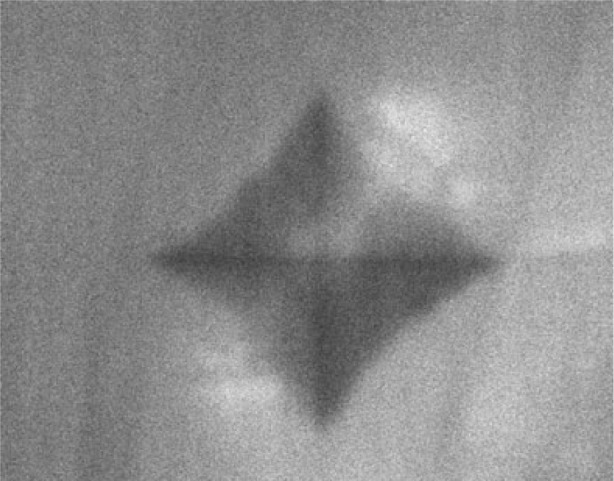
CL image around a 500 g load Vickers indentation of a natural massive ZnS crystal.

The creation of recombination centers by the static indentation are also shown on the CL image in Fig. 18 for a massive ZnS crystal. These observations are consistent with CL microscopy and spectrometry data reported by Boyarskaya et al. [21] for the case of an indented MgO single crystal. These authors showed that the variations in CL around the indent are associated with a variation of the intensity of the UV CL peak as a function of the distance from the indentation crater. In addition Boyarskaya et al. [21] associated the UV emission peak with the formation of F^+^ centers, i.e., an anion vacancy with a trapped electron.

**Fig. 19:**
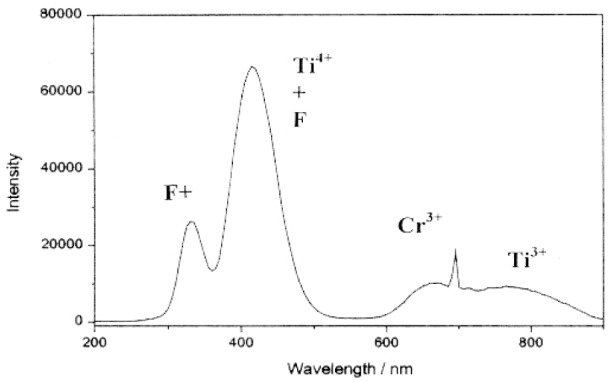
CL emission spectrum measured from an un-doped *α*-alumina single crystal with a 25 keV incident electron energy irradiating a 300 μm × 300 μm area.

The creation of F^+^ and F (an anion vacancy with two trapped electrons) centers resulting from the cutting action of abrasives is clearly illustrated for the case of a diamond polished *α*-alumina un-doped single crystal that has a characteristic CL emission spectrum shown in Fig. 19.

**Fig. 20:**
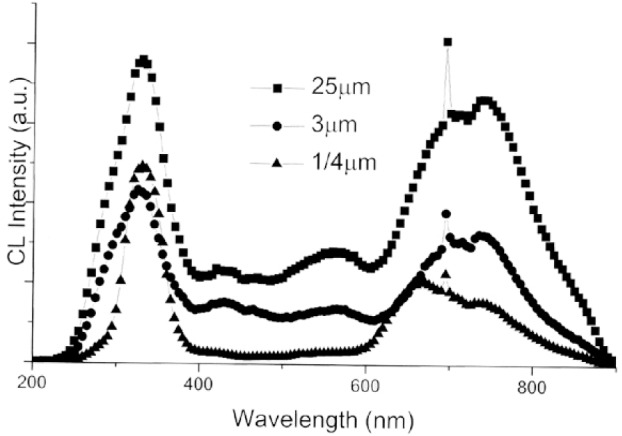
Effect of abrasive wear on the CL emission properties of an *α*-alumina crystal as a function of the abrasive grain size.

Even in non intentionally doped alumina crystals, small amounts of Cr are frequently present and the Cr^3+^ impurity ions are identified on the CL emission spectrum by a narrow line at 694 nm. Titanium is also a frequent impurity and the presence of Ti^3+^ ions result in a CL emission band near 720 nm. As shown in Fig. 20, several emission bands occur in the UV region. The emission band at 320 nm corresponds to the presence of F^+^ centers formed from oxygen vacancies. The emission near 430 nm is attributed to F centers, however, as discussed by Phillips et al. [22] , the 430 nm emission band is complex resulting also from a negatively charged center (aluminum vacancy V_Al_ center) and/or from the presence of Ti^4+^ ions. The shape and the relative intensities of the CL emission bands for *α*-alumina single crystals vary as a function of the grain size of the abrasives used for polishing as illustrated in Fig. 20.

**Fig. 21:**
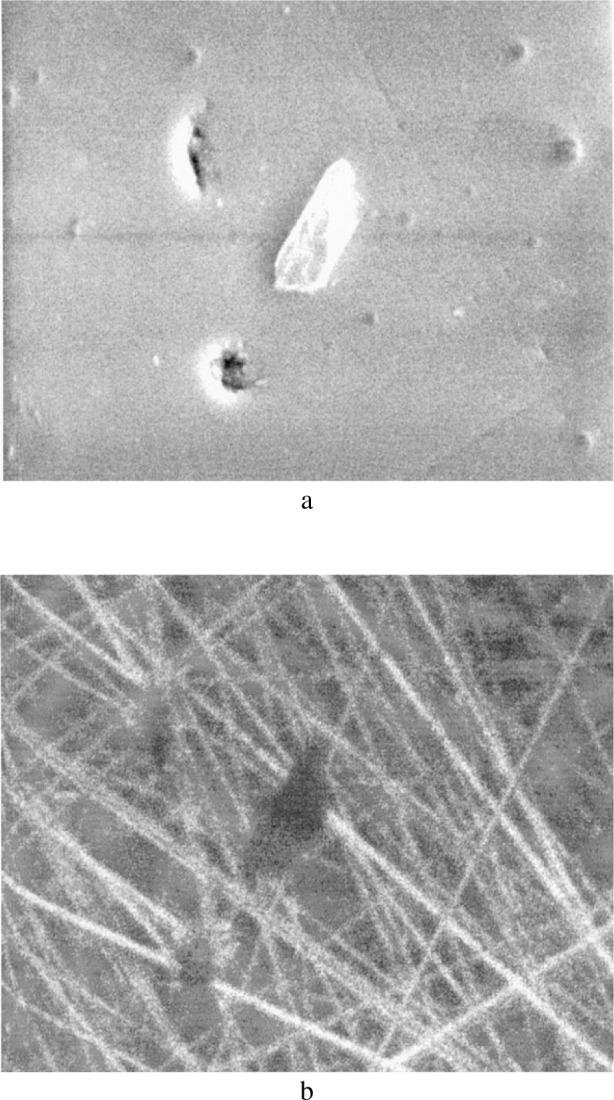
Subsurface damage resulting from the abrasive wear of an a-alumina crystal (a) secondary electron image and (b) selected wavelength CL image at λ = 320 nm corresponding to F^+^ centers.

The secondary electron image of an *α*-alumina crystal in Fig. 21a shows the presence of remaining holes and dust particles, but deep scratches resulting from the polishing with 0.25 μm diamond grains on a nylon pad are not visible. The CL image in Fig. 21b at the 320 nm selected wavelength corresponding to the characteristic emission of F^+^ centers reveals a high density of bright lines. These bright line are “ghosts” of subsurface scratches induced by the abrasives. By varying the energy of the incident electrons networks of F^+^ centers at different depths below the specimen surface can be observed.

## 5. Implications of the Polishing Procedure in Quantitative X-Ray Microanalysis

### 5.1 Layered Structures Resulting From Polishing

A diamond polished (0.25 μm abrasives) massive chalcopyrite specimen was analyzed to compare to the same material which was submitted to a final polishing step on a soft pad with chromic oxide dispersed in water. The experimental concentrations were corrected assuming a constant composition as a function of depth below the specimen surface.

Results in Table 3 illustrate the sensitivity of EPMA data to surface composition. In practice, in order to minimize the effect of differences in surface compositions the analyzed specimen and reference materials must be rigorously prepared according to the same polishing procedure. Since surface composition changes may still occur during storage in the air for a prolonged time, EPMA analyses must be performed immediately after polishing.

Point analyses at the surface of freshly polished chalcopyrite inclusions in contact or isolated from the silver sulfide matrix were performed at 8 keV and 15 keV incident beam energy successively. The Ag experimental concentrations were processed assuming that the Ag is homogeneously distributed in the chalcopyrite inclusions. Results led to a Ag concentration equal to 0.85 % when measured with a 8 keV incident electron energy and a value equal to 0.3 % when the incident energy was increased to 15 keV. These results express a non uniform depth distribution of the Ag concentrations but do not allow us to conclude whether a Ag surface contaminant is only present at the surface or is simultaneously present in the bulk of the chalcopyrite inclusions.

The experimental data were then processed assuming successively that the measured Ag intensity originated from (i) the bulk chalcopyrite and (ii) a surface Ag bearing compound as discussed by Rémond et al. [23].

Results are shown in Table 4 for the case of tarnished inclusions in contact with the Ag2S matrix kept in the air for a long time. For the three incident energy values, the calculated Ag concentrations are in a good agreement with the measured concentrations using the same thickness of the Ag bearing surface layer for the calculations. For the inclusions isolated from the matrix, as shown in Table 5, a less satisfactorily agreement is observed between data derived from experiments at 30 keV. The difference between experimental and calculated data from the 30 keV experiment suggests that Ag should be simultaneously present either as a surface contaminant or as a bulk impurity within the chalcopyrite inclusions.

In absence of an accurate knowledge of the composition and texture of the surface compounds, any attempt in processing the EPMA data accounting for both Ag locations, surface and bulk, should be purely speculative. Calculations showed that these statistical limits of detection lead to a measured Ag intensity equal to that resulting from a 0.2 nm thick pure Ag layer at the surface of the chalcopyrite.

### 5.2 Electrostatic Charging Phenomena

Charging is a major concern when studying strongly insulating materials with electron beam based techniques. The existence of an inside trapped charge can easily be visualized by measuring the Duane-Hunt limit (DHL) on an energy dispersive x-ray spectrum (EDS) from an uncoated insulating material as reported by Bastin [24] and by Rémond et al. [25]. The DHL is the maximum energy of the emitted x-ray photons which in absence of charging equals the value of the incident electron energy.

**Fig. 22:**
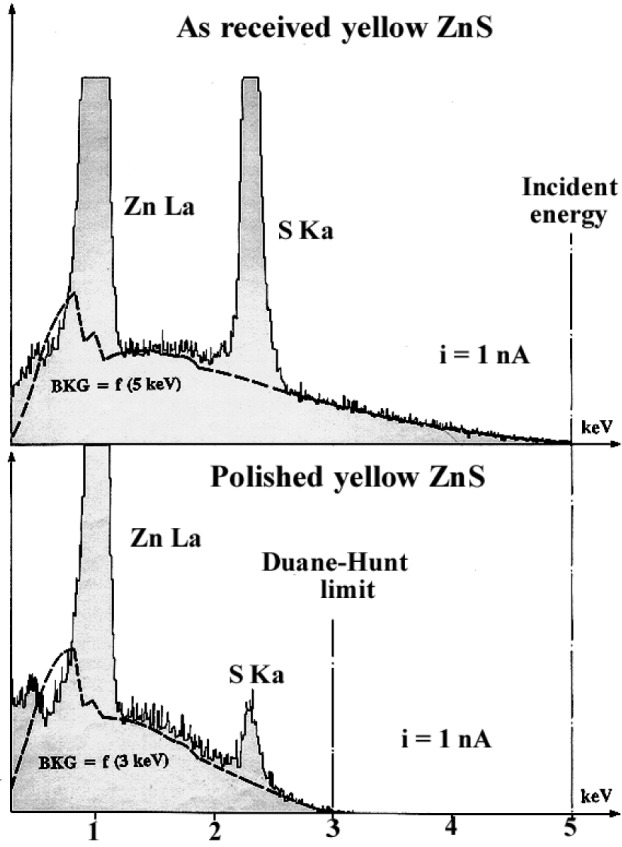
EDS spectra measured from an uncoated ZnS polycrystalline specimen, in the “as received form” and after mechanical polishing according to the procedure B in Table 2, irradiated in vacuum with a 5 keV incident energy. The calculated distribution of the x-ray continuous emission is performed using the energy of the Duane-Hunt limit as landing energy.

Changes in the DHL positions as a function of the specimen preparation are shown in Fig. 22 for the case of two synthetic ZnS polycrystalline specimens. An EDS spectrum (Fig. 22a) was measured with a 5 keV incident energy from the initial polished “as received” specimen surface (polishing procedure not provided by the specimen manufacturer). No charging was observed as indicated by the DHL occurring at the 5 keV incident energy value. In addition the shape of the observed continuous distribution was correctly analytically described. The specimen was mechanically polished according to the procedure B described in Table 2. As shown in Fig. 22b, the DHL occurs at 3 keV, indicating charging. In addition, the continuous x-ray emission was satisfactorily described assuming that the landing energy of the incident electrons was 3 keV instead of the nominal 5 keV value, indicating a 2 keV reducing potential in the specimen chamber of the SEM.

**Fig. 23:**
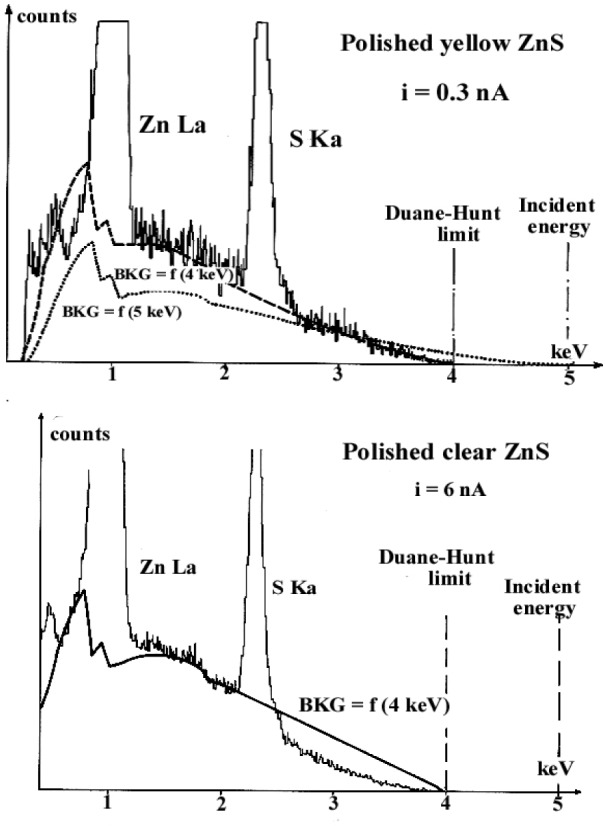
EDS spectra measured from two uncoated ZnS polycrystalline specimens polished according to the procedure B in Table 2, irradiated in vacuum with a 5 keV incident energy. The incident beam current is adjusted in order to have the DHL value for both specimens. The continuous x-ray emission distributions are performed using the energy of the Duane-Hunt limit as landing energy.

The two synthetic ZnS specimens, polished according to the procedure B in Table 2 were analyzed with a 5 keV incident electron beam. The incident beam current was adjusted in order to obtain the same DHL position at 4 keV for both specimens (Fig. 23). For the polished yellow specimen (Fig. 23a), the continuous emission was correctly modeled assuming a 4 keV landing energy. For of the clear specimen (Fig. 23b), it was not possible to fit the experimental continuous emission with calculated data.

**Fig. 24:**
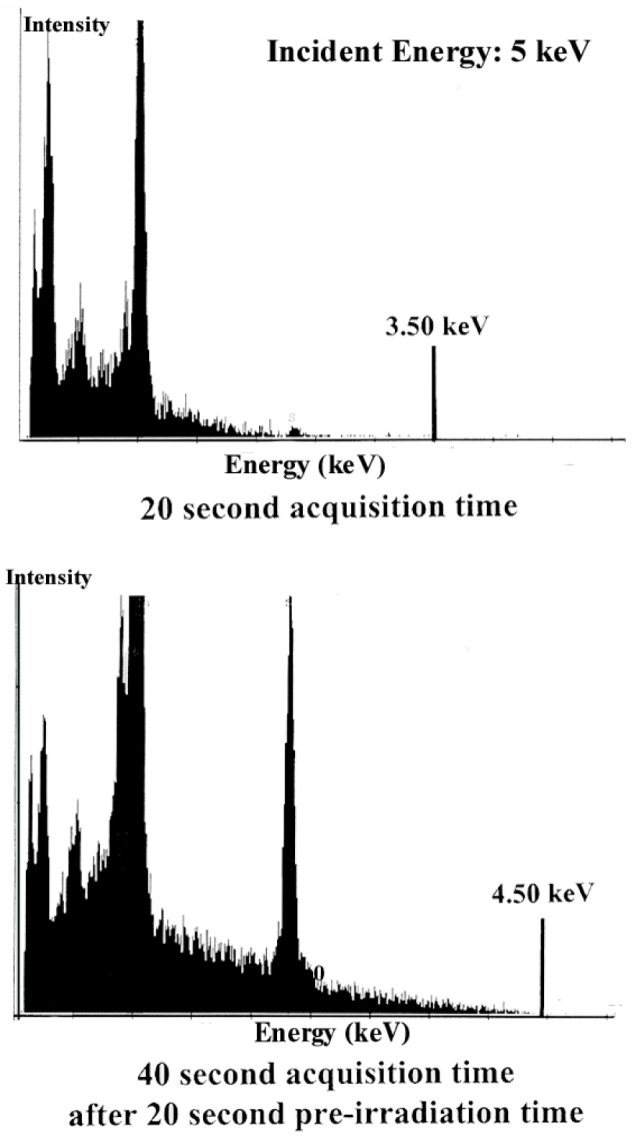
EDS spectra measured for two acquisition times from two uncoated ZnS polycrystalline specimens polished according to the procedure B in Table 2, irradiated in vacuum with a 5 keV inciden energy.

In order to explain the deviation between the observed and calculated continuous emission, a dynamic charging effect as a function of the irradiation conditions must be considered. This dynamical effect is illustrated by the EDS spectra (Fig. 24) measured from an uncoated colloidal silica polished ZnS specimen analyzed in VP-SEM (0.1 mbar residual air pressure and 5 keV incident energy). A first EDS spectrum was recorded with 20 s acquisition time (Fig. 24a). The DHL position was found at 3.5 keV indicating that during the acquisition time the lowest negative retarding potential was 1.5 keV. A second spectrum was measured from the same location with a 40 s acquisition time and the Duane-Hunt limit was located at 4.5 keV (Fig. 24b) indicating a lower charging effect than that observed during the first irradiation.

The observed spectrum stored in the multichannel analyzer after a predetermined acquisition time is the sum of individual spectra whose Duane-Hunt limit is varying as a function of time. The observed DHL corresponds to the highest value reached during the experiment. The resulting EDS spectrum is equivalent to a sum of spectra acquired with different excitation conditions. Consequently the shape of the continuous emission cannot be described assuming a constant incident energy and a deviation between the measured and the calculated continuous emission indicates a variation of the reduction potential during the irradiation time.

**Fig. 25:**
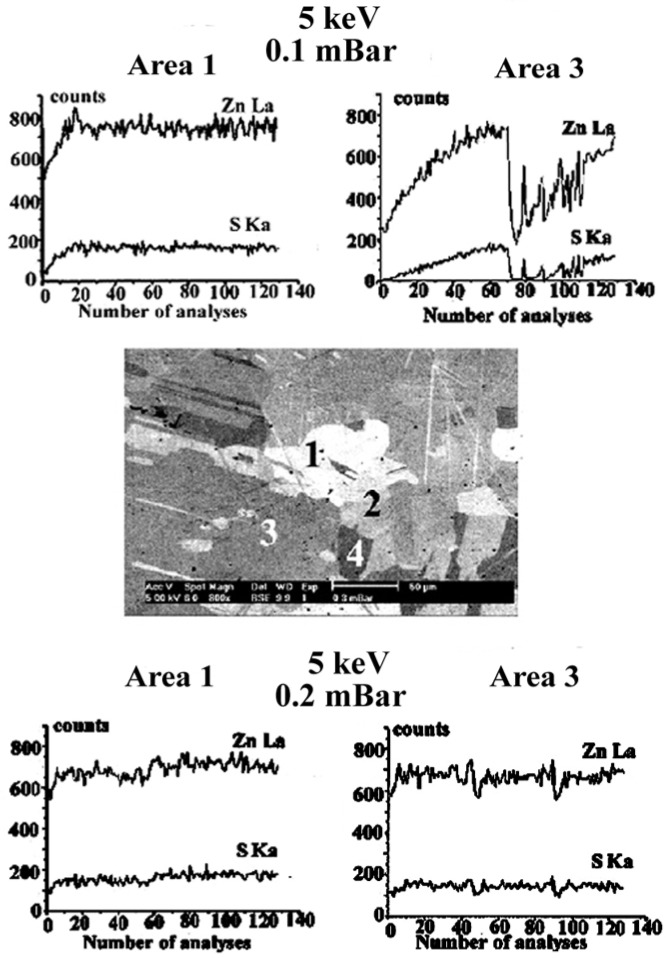
Kinetics of the ZnL*α* and SK*α* intensities measured from a colloidal polished specimen in a VP-SEM.

Another way to observe the variations of the trapped charge as a function of irradiation time is to study the x-ray intensity variations as reported by Rémond et al. [26]. The ZnL*α* and SK*α* intensities were measured from the four locations shown on the backscattered electron image in Fig. 25. About 150 spot mode measurements were performed with a 2 s sampling time, a 5 keV incident energy and a 0.1 mbar residual air pressure in the specimen chamber of the VP-SEM. The variation of the x-ray intensities for areas 1 and 4 and 2 and 3, respectively, were similar. As shown in Fig. 25, for the case of areas 1 and 3, the x-ray intensities increased with irradiation time before reaching a steady state. For points 2 and 3, a breakdown is observed after the steady state was reached resulting in very rapid variations of the x-ray intensities. No such variations were observed for the case of areas 1 and 4.

For the four analyzed areas, no SK*α* signal was observed at the beginning of the irradiation, indicating a strong negative charge reducing the landing energy below the SK excitation threshold. The initial negative surface charge is consistent with the result in Fig. 24, which exhibits a DHL shift to 3.5 keV after a 20 s acquisition time. The DHL position was shifted towards the incident energy value when the acquisition time was prolonged, consistent with the observed increase of the x-ray intensities. When the steady state is reached the trapped charge d*Q*/d*t* is equal to zero but, *Q* is different from zero. When the residual air pressure is increased to 0.2 mbar, the steady state is reached more quickly and dielectric breakdown is no longer observable. The increase of the x-ray signal is due to a decrease in the charging effect when the irradiation time is prolonged indicating that the release of trapped charges is not immediately compensated by the incoming positive ions at the specimen surface.

A possible explanation for the dielectric breakdown observed at area 3 in Fig. 25 for the case of a 0.1 mbar residual gas pressure is that of an incomplete surface charge compensation. The incident electron beam can be shifted from its initial position to a non- irradiated area which is strongly negatively charged resulting in a rapid decrease of the intensity signals.

Although trapping defects may intrinsically exist in the ZnS crystals, it is apparent that the final polishing with colloidal silica is not sufficient to remove all surface and subsurface damage since no charging was observed from EDS analysis from a freshly fractured surface of the same specimen even when EDS analyses were performed in high vacuum.

In practice, for quantitative microanalysis with the EPMA, the specimen surface is coated with a grounded thin metallic film. Changes in the DHL position are no longer observed since the surface potential is always zero. Bulk charging still occurs but the electrons removed from traps return to the ground potential through the coating film and the electric field does not penetrate into the vacuum as it is pinned at the conductive coating. The internal electrical field resulting from the bulk intrinsic defects and those created near the surface during polishing will maximize at the interface between the surface coating and the specimen surface [27]. As a result, the depth ionization distribution is modified as a function of the local tapped charge in the interaction volume as illustrated for the case of quantitative analysis of alumina crystals.

Single and polycrystalline *α*-alumina crystals were also prepared using both mechanical and CMP final polishing procedures. Again, the colloidal silica polishing revealed the grain structure of the polycrystalline specimens, which was not visible after the final mechanical polishing with fine diamond paste. The specimens were carbon coated for quantitative analysis with the EPMA. The AlK*α* and OK*α* intensities were measured from the mechanically polished (0.25 μm diamond) surfaces and compared to those measured from the silica polished surfaces of the same materials. Results are shown in Table 6 and Table 7. These results show that (1) the intensity ratios for the AlK*α* and OK*α* for the same specimen depend on the final polishing procedure used, (2) the intensity ratios vary with the incident energy and tend to unity when the analyzed depth increases and (3) for a given final polishing procedure, the intensity ratios for a polycrystalline specimen analyzed in respect to a massive specimen of same composition vary with the incident energy.

## 6. Discussion

Polishing with abrasive particles is a complex operation that involves a set of tribological parameters that do not only depend on the chemical and physical properties of the specimen to be polished.

In practice, the abrasive wear will depend on the distribution of the shape of the abrasives and on the height of the particles with respect to the altitude of a mean cutting plane. For an accurate modeling of the abrasive wear it is thus important to account for i) the number of particles per unit area, ii) the mean diameter of the abrasives, the mean difference in height between the grains and iii) the distance between each grains. In addition to the purely mechanical approach, the mechanisms of abrasive wear also depend on the nature of the fluid used as a carrier of the abrasives. The liquid has the effect of improving the wetting of the abrasives and the surface to be polished. Most frequently, pure water or water with additives are used. The presence of surfactants as additives aims to decrease the superficial energy of the materials which results in a change of the interfacial shear stress.

**Fig. 26:**
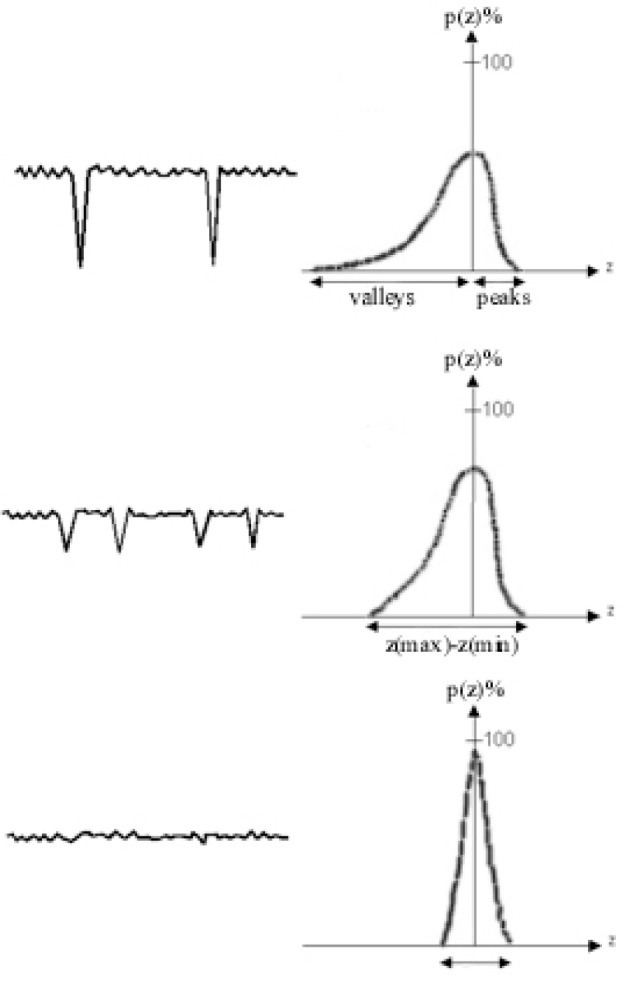
Schematic evolution of the distribution, *p* (*z*) of the heights, *z*, of asperities resulting from polishing with decreasing abrasive grain size.

**Fig. 27:**
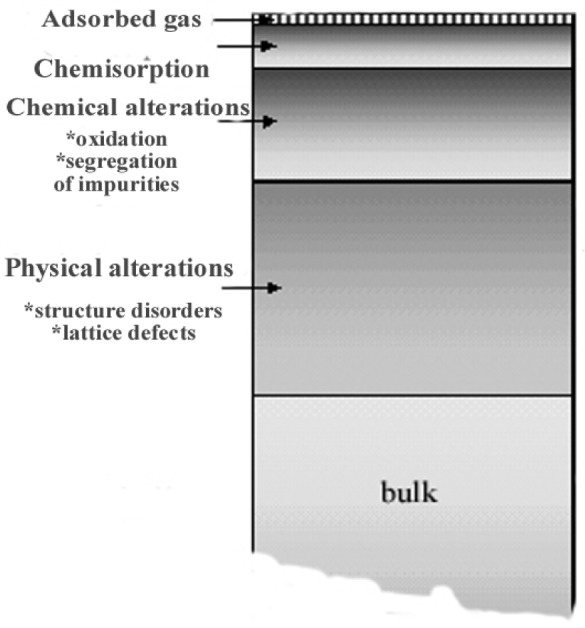
Schematic illustration of physical and chemical modifications as a function of depth of a polished material.

In order to correlate the quantitative data derived from x-ray spectrometry with the polishing stages it is important to recall that:

1. the polishing results mostly from abrasive mechanisms,
2. the polishing techniques involving abrasive particles of decreasing grain size result in a modification of the surface roughness of the specimen surface as illustrated in Fig. 26 and,
3. the abrasive wear result in modifications f the physical and chemical properties as summarized in Fig. 27, where these modifications are dependent on the specimen to be polished.

When an optically polished surface is obtained in order to satisfy the geometrical requirements for quantitative x-ray microanalysis, a residual topography at the nanometer scale still exists. More attention should be given to the effect of nano-roughness on the absorption factor for soft x-ray emission characterized by shallow escape depths. The recent literature on micro-analysis indicates an increasing interest in microanalysis of rough surfaces [28] and these investigations at the microscopic scale should be extended to the nanometer scale.

Most EPMA laboratories use a similar three stage polishing procedure as summarized in Table 1, but the nature of polishing materials and equipment may vary from one laboratory to another. When the optically polished condition is satisfied, the quantitative x-ray data vary as a function of the choice of the experimental and instrumental polishing parameters as illustrated in this study using the two polishing procedures described in Table 2.

The formation of an amorphous surface layer resulting from the abrasive wear was first suggested by Beilby [29]. Recent characterization of finely polished surfaces of semiconductors supports the presence of an amorphous surface compound, as well as subsurface lattice disorder as illustrated by Lucca and Maggiore [30] for the case of II—VI semiconductors analyzed by ion channeling. These authors showed that the thickness of the amorphous layer and the damage depth depend on the material but have lower values for a CMP polishing than for a purely mechanical polishing with quarter micron diamond abrasives. In this study, the presence of a heavily damaged surface was observed for the case of the polycrystalline ZnS specimens and the crystallographic contrast was observed on back-scattered electron image after the damaged layer was removed by combining mechanical polishing with chemical reactions.

As the roughness of the polished surface decreases, the contact area and the friction with the polishing substrate increase. The resulting thermal effects may induce surface chemical reactions that are accelerated by an electrochemical effect due to the electrical charges created during the abrasive wear mechanism. The surface of mechanically polished metals rapidly oxidizes. The thickness of the oxidized layer may vary from a few nanometers to some hundreds of nanometers depending on the nature of the metal and the oxidation kinetics. Surface oxidation of natural sulfide minerals have been reported for the case of pyrite [31] that is illustrated in the study with chalcopyrite specimens described here.

According to Maurel [32], the mechanisms of thermal oxidation in air of natural sulfide minerals are accompanied by volatilization of S with an increase of the metal concentration at the surface with formation of an oxide. Such a thermal effect may be invoked to explain the formation of a surface iron oxide layer at the surface of the massive chalcopyrite specimen polished with diamond abrasives on a hard nylon polishing pad. During this polishing procedure, electrical charges are produced due to bond breaking resulting in the emission of exo-electrons, while simultaneously the local temperature increase is probably high. Polishing on a soft pad with very small particles dispersed in a large quantity of water tends to minimize the thermal increase and the surface compounds are progressively removed.

For inclusions, surface contaminants can also contain some species from the adjacent materials as illustrated for the case of chalcopyrite inclusions in a silver sulfide matrix. Silver sulfide is easily decomposed by light and thermal effects as reported by Stephens [33]. According to this author, light alone leads to the decomposition of the silver sulfide while light illumination combined with a temperature increase provokes the sublimation of sulfur with the formation of metallic silver at the surface. The presence of a silver bearing compound on top of the inclusions in the silver sulfide may result from a smearing effect of wear debris of the matrix occurring either directly by mechanical transfer to the adjacent surfaces or indirectly by the polishing disc contaminated by wear debris formed during the polishing procedure or a previous one. The surface reaction products can also be formed by thermal and/or electrical lateral assisted diffusion of silver bearing species through the residual water on the surface. This mechanism results in a continuous modification of the surface composition after polishing depending on the storage conditions of the polished surfaces. The example of the inclusions in the silver matrix is an extreme situation where tarnishing and contamination are observable immediately after polishing. In many other instances such effects only occur after a long period of storage in the atmosphere of the polished specimens as reported by Rémond et al. [17].

For polycrystalline metals, the oxidized layer develops on top of a structurally damaged zone characterized by strain hardening and preferential orientation of the crystallites. Structural changes have been observed around the Vickers indentations for different applied loads at the surface of the polycrystalline ZnS specimens. The grains boundaries are made visible around the indents indicating a de-cohesion of the crystallites. In insulating materials, stresses and strains may also be responsible for the creation of lattice defects, some of them acting as electron traps responsible for electrostatic phenomena during electron irradiation.

The most commonly used indicator of charging is to measure the position of the DHL, i.e., the maximum energy of emitted x-ray photons on an EDS spectrum of an uncoated insulator placed in vacuum or in residual gas pressure. However, the DHL position depends on the incident dose and varies as a function of the irradiation time. The DHL position also depends on instrumental factors (such as grounded stage geometry and position of biased detector) modifying the geometrical distribution of the equipotential lines in the specimen chamber and, the position of the DHL is thus not an intrinsic property of the specimen. Furthermore, for a specimen coated with a grounded surface conductive layer, as used for quantitative x-ray microanalysis with the EPMA, the potential of the surface is permanently equal to zero and consequently no shift in the DHL position will be observed. In practice, DHL measurements from uncoated insulators that are performed in the SEM or ESEM cannot be simply extrapolated to the same specimen placed in the EPMA conditions.

Mineral materials are frequently heterogeneous and the nature, the spatial distribution and the density of electron traps vary at the micrometer scale. In order to predict and correct the effects of intense internal electrical fields developing in the specimen on the depth distribution of ionizations it is necessary to determine the trapped charge at exactly the same electron beam position as that selected for x-ray microanalysis. In some instances, the presence of energy levels associated with impurities or point lattice defects can be obtained from luminescence studies (CL spectrometry and microscopy) as illustrated in this study for the case of F^+^ centers along the scratches resulting from the abrasive wear of alumina crystals. Jonnard et al. [34] showed that the energy levels associated with the F^+^ centers are responsible for a weak energy satellite occurring on the high-energy side of the Al Kβ emission peak. However, such investigations can only be performed in some particular situations and cannot be used as general signature of defects acting as electron traps.

Each successive stage of polishing with abrasives of decreasing grain size as described in Tables 1 and 2, aims to remove the damage created by the previous polishing conditions but each step creates its own damage. The thickness of the damaged layer can be minimized but it is not possible to completely prevent against surface damages by mechanical and/or mechanical-chemical polishing. As a result, a thin surface layer on top of the substrate is often difficult to detect by optical observation because it generally results in a decrease of reflectivity without an observable change in color.

Surface contaminants and compounds forming a layered structure on top of the bulk material or a continuous in-depth gradient of composition will affect the accuracy of the quantitative data. In order to minimize the effect of surface composition on quantitative x-ray data representative of the bulk composition, both the reference and the unknown specimens must be rigorously polished using the same procedure. To prevent surface composition changes during storage of the polished specimens in the air for a long time, EPMA analyses must be performed as soon as possible after the polishing procedure is applied.

In practice, the easiest way to detect thin surface contaminants is to verify that quantitative measurements obtained at different electron beam energies are comparable. In the presence of quantitative data varying as a function of the incident energy, the specimen must be treated as a layered specimen. Surface sensitive techniques must be used to model the depth composition.

Charging phenomena will also result in a dependence of quantitative data as a function of the excitation conditions. The spread of quantitative data as a function of the choice of the reference compounds may also be an indicator of the presence of charging phenomena. However at the present time, there is no analytical solution to the problem of electrostatic charging and in practice, these effects can be minimized by: (1) scanning the beam over a large area (2) using low incident energy and a low beam current and (3) choosing the standards in order to have similar effects as those occurring for the case of the unknown. In practice, the structural and textural properties of both the unknown and the standards must be considered and not just their chemical composition in order to have similar charging effects in both materials. This requirement is more particularly important when soft x-ray photons are analyzed. However, the selection of the standards for a particular analyzed material cannot be obtained from the macroscopic properties of the compounds since the charging phenomena result from the presence of electron traps whose density and spatial distribution vary at the microscopic scale. More investigations are needed in order to provide an indicator to select the appropriate reference compounds.

## 7. Conclusion

All EPMA laboratories use similar polishing procedures in order to obtain optically polished surfaces. However, varying the nature of polishing materials and equipment may result in different surface and subsurface chemical, physical and textural damage even in materials exhibiting similar geometrical surface properties. Although these perturbations affect a shallow depth below the specimen surface, they are sufficient to alter the accuracy of quantitative x-ray analyses with the EPMA.

Since the unknown and the reference specimens are rigorously prepared according to the same procedure, a spread of the quantitative x-ray data as a function of the excitation conditions is usually observed, at least for the case of the analysis of the major constituents and for high incident x-ray photons. However, remaining surface and subsurface damage may alter the accuracy of the quantitative data for the case of elements present at trace levels or for the case of analyses performed at low incident energies or when soft x-ray photons are analyzed. Textural and lattice disorders resulting from abrasive wear may be responsible for the development of internal electrical fields in strongly insulating materials leading to charging phenomena. These effects are difficult to predict. The procedure used for the specimen preparation should always be added to the presentation of the experimental conditions and should be more particularly mentioned in the strategy used to prepare reference materials for x-ray microanalysis.

## Figures and Tables

**Table 1:** Usual polishing procedures

Polishing stage	Metallic alloys	Mineral materials
1 Coarse polishing	Emery paper of decreasing grade (bound abrasive process).	Abrasive slurries on cast iron or glass plate (loose abrasive process).
2 Polishing	Diamond or alumina grains (loose abrasive process).	Diamond grains fixed on the metallic disc (bound abrasive process).
3 Finishing	Glazing on cloth, electrolytic or chemical polishing.	Polishing with alumina on a cloth (loose abrasive process) and/or, mechanical + chemical polishing.

**Table 2:** Polishing procedures used in the present study

Operation	Procedure A	Procedure B
Coarse polishing	25 μm diamond paste on a cast iron plate.	25 m SiC slurry on a glass plate.
Intermediate polishing	6 μm, 3 μm, and 1 μm diamond paste on a tin plate. Water as lubricant.	6 μm, 3 μm, and 1 μm diamond grains on a hard plate covered with an aluminum foil. Silicone oil as lubricant.
Final polishing	0.25 μm diamond paste on a hard plate covered with a napless tissue. Water as lubricant.	1) 0.5 μm diamond grains on a hard plate covered with nylon. Silicone oil as lubricant.
		2) 20 nm alumina dispersed in distilled water on a soft pad.
Optional	Colloidal silica dispersed in a ph = 10 solution on a metallic plate covered with a soft pad.	

**Table 3:** EPMA quantitative analyses of a diamond polished chalcopyrite (CuFeS_2_) specimen using the same chromic oxide polished chalcopyrite specimen concentrations in mass fraction × 100

Analyzed x-ray lines	Incident energy (keV)		
	15	20	30
S K*α*	34.3	34.7	33.4
Fe K*α*	26.7	30.0	30.1
Cu K*α*	32.6	33.3	33.4
∑	93.6	98.0	96.9

**Table 4:** Calculated Ag concentrations assuming 0.30 mass fraction Ag in a 15 nm thick layer on top of tarnished chalcopyrite inclusions in contact with a Ag2S matrix (Rémond et al. 1984). Concentrations in mass fraction × 100

Incident energy keV	Experimental concentration	Calculated bulk concentration	Calculated surface concentration
8	3.92	4.60	3.69
15	1.08	1.37	1.08
30	0.43	0.65	0.45

*CA* (*ρz*) = A* exp – (*δρz*)^2^

A* = 30 %, *δ* = 6.6 104 cm^2^ g^–1^.

**Table 5:** Calculated Ag concentrations assuming 0.30 mass fraction Ag in a 2.5 nm thick layer on top of tarnished chalcopyrite inclusions isolated from the Ag2S matrix (Rémond et al. 1984). Concentrations in mass fraction ×100

Incident energy keV	Experimental concentration	Calculated bulk concentration	Calculated surface concentration
8	0.60	0.80	0.61
15	0.16	0.21	0.18
30	0.13	0.20	0.08

*CA* (*ρz*) = A* exp – (*δρz*)^2^

A* = 30 %, *δ* = 4 105 cm^2^ g^–1^.

**Table 6:** AlK*α* and OK*α* intensities measured as a function of the incident energy, from diamond polished alumina specimens, relative to the AlK*α* and OK*α* intensities, respectively measured from the same specimens polished with colloidal silica particles

	*I*(AlK*α*, diamond polished) *I*(AlK*α*, silica polished)		
Specimen	5 keV	15 keV	25 keV
Natural single crystal	1.242	1.016	1.008
Polycrystalline	1.136	1.007	1.009

	*I*(OK*α*, diamond polished) *I*(OK*α*, silica polished)		

Natural single crystal	1.271	1.136	1.139
Polycrystalline	1.189	1.091	1.086

**Table 7:** AlK*α* intensity measured as a function of the incident energy from a polycrystalline alumina relative to that measured from a single alumina crystal. The specimens are mechanically polished with diamond abrasives and with colloidal silica (mechanical-chemical polishing) successively

	*I*(AlK*α*, polycrystalline) *I*(AlK*α*, single crystal)		
Polishing procedure	5 keV	15 keV	25 keV
0.25 μm diamond	0.970	1.000	0.998
Colloidal silica	1.058	0.993	0.980
